# Hypothalamic neuroimmune remodeling as an immunometabolic bridge between childhood obesity and earlier pubertal onset

**DOI:** 10.3389/fimmu.2026.1932785

**Published:** 2026-08-25

**Authors:** Ping Liu, Yue Chen, Yujing Zhang, Yanhui Peng, Enze Lei, Jianzhong Liu

**Affiliations:** 1 Hubei University of Chinese Medicine, Wuhan, Hubei, China; 2 Hubei Shizhen Laboratory, Wuhan, Hubei, China; 3 Jianghan University Affiliated Hubei Third People’s Hospital, Wuhan, Hubei, China; 4 Hubei Provincial Hospital of Traditional Chinese Medicine, Affiliated Hospital of Hubei University of Chinese Medicine, Wuhan, Hubei, China

**Keywords:** central precocious puberty, childhood obesity, HPG axis, hypothalamic neuroimmune remodeling, kisspeptin–GnRH network, metaflammation, pubertal onset

## Abstract

While the link between childhood obesity and central precocious puberty (CPP) is well documented, the immune-mediated mechanisms that may precipitate earlier pubertal onset remain incompletely understood. This review proposes hypothalamic neuroimmune remodeling as a candidate immunometabolic bridge through which chronic low-grade peripheral inflammation may influence the timing of hypothalamic-pituitary-gonadal (HPG) axis activation. In obesity, systemic immune activation is primarily by adipose tissue macrophage infiltration, lipotoxic signaling, and gut dysbiosis. Peripheral inflammatory signals may reach the central nervous system through the arcuate nucleus-median eminence complex, disruption of the blood-brain barrier, and immune-mediated gut-brain pathways. Within the hypothalamus, these signals may elicit local neuroimmune responses. Glial cells, particularly microglia, astrocytes, and tanycytes, undergo inflammatory activation and release cytokines and lipid mediators, including IL-1β, TNF-α, and prostaglandin E2. This localized neuroimmune response may alter the excitatory milieu and synaptic connectivity of the kisspeptin-GnRH network, thereby lowering the threshold for HPG axis activation. This review systematically synthesizes current evidence on this immune-endocrine crosstalk and describes how peripheral immune signals may remodel the hypothalamic microenvironment and alter pubertal timing. We further discuss the translational potential of targeting specific neuroimmune pathways for biomarker discovery and the development of novel immunomodulatory interventions for CPP. Although neuroimmune remodeling represents a plausible mechanism underlying obesity-associated pubertal advancement, its causal role requires further validation through targeted mechanistic studies and longitudinal immunophenotyping.

## 1 Introduction

Earlier pubertal onset and precocious puberty, particularly central precocious puberty (CPP), have become major concerns in child development and public health (1, 2). CPP is characterized by premature activation of the hypothalamic-pituitary-gonadal (HPG) axis. Epidemiological evidence indicates that the global prevalence of precocious puberty is approximately 7.87% among girls and 3.98% among boys, with higher rates among children with obesity (3). Overweight/obesity and elevated body mass index (BMI) are closely associated with CPP and earlier pubertal onset, particularly among girls (4, 5). These findings suggest that metabolic disturbances during childhood may contribute to the regulation of reproductive axis maturation, although excess adiposity alone is unlikely to determine pubertal timing.

Pubertal onset is governed by hypothalamic gonadotropin-releasing hormone (GnRH) neurons and their upstream regulatory networks, which integrate diverse nutritional, metabolic, immune, environmental, and sex steroid signals (6). In the context of diets high in fat and sugar, overnutrition, and insufficient physical activity, childhood overweight/obesity is frequently accompanied by insulin resistance, adipokine dysregulation, and chronic low-grade inflammation, a state known as metaflammation (7–9). Conventional “energy reserve” and “leptin threshold” theories may partly explain the association between nutritional status and pubertal onset but cannot fully account for the heterogeneity of obesity-associated pubertal advancement across sex, obesity severity, and clinical phenotypes (10, 11). How obesity-associated metabolic disturbances are sensed by the central neuroendocrine system and translated into premature HPG axis activation therefore remains unresolved.

Emerging evidence indicates that the hypothalamus functions not only as a higher-order center for energy homeostasis and reproductive axis regulation but also as a key immunometabolic interface through which peripheral metabolic and inflammatory signals influence neuroendocrine networks (12, 13). In the setting of obesity or high-fat diet exposure, inflammatory cytokines, adipokines, free fatty acids, and lipopolysaccharide (LPS) may alter the hypothalamic microenvironment through changes in BBB permeability, circumventricular organs, and gut-brain axis pathways (14–16). These stimuli may trigger reactive changes in microglia, astrocytes, tanycytes, and vascular-associated cells. They may also activate pathways involving Toll-like receptor 4/nuclear factor-κB (TLR4/NF-κB), the NOD-like receptor family pyrin domain-containing 3 (NLRP3) inflammasome, and prostaglandins, thereby altering kisspeptin inputs, GnRH neuronal excitability, and synaptic plasticity (17–20). Collectively, these findings support a central hypothesis: hypothalamic neuroimmune remodeling may constitute a candidate immunometabolic bridge linking childhood obesity to earlier pubertal onset.

Previous studies have separately examined the epidemiological association between childhood obesity and CPP (4, 21), the effects of metabolic signals such as adipokines and insulin on the HPG axis (22, 23), and obesity-induced hypothalamic inflammatory responses (9, 24). However, a unified framework integrating the entire sequence from peripheral metabolic dysfunction to central immune activation, neuroendocrine network remodeling, and earlier pubertal onset remains lacking. Accordingly, this review begins with peripheral metaflammation and examines how these inflammatory signals are transmitted to the hypothalamus through multiple pathways, remodel the neuroimmune microenvironment comprising neurons and glial cells, and ultimately influence the timing of kisspeptin-GnRH network activity and HPG axis activation. By integrating these processes, this review aims to advance mechanistic understanding and inform risk identification and metabolic–immune interventions for obesity-associated earlier pubertal onset in children.

## 2 Neuroendocrine regulation of pubertal onset and its relevance to metabolic cues

### 2.1 Neuroendocrine control of pubertal onset

The central event in pubertal onset is reactivation of the HPG axis, marked by increased pulsatile hypothalamic secretion of GnRH. GnRH reaches gonadotrophs in the anterior pituitary through the hypophyseal portal system and stimulates the secretion of gonadotropins, including luteinizing hormone (LH) and follicle-stimulating hormone (FSH). These hormones subsequently activate gonadal steroidogenesis, including the production of estradiol, progesterone, and testosterone, and promote pubertal development (25). Although GnRH neuronal activation is central to pubertal onset, the upstream regulatory networks and mechanisms responsible for the developmentally timed release of the GnRH system from inhibitory control remain incompletely understood (26). Current evidence indicates that pubertal onset is not triggered by a single factor. Instead, it is coordinately regulated by genetic and epigenetic programs, hypothalamic neuronal populations, neuropeptides, nutritional status and metabolic signals, sex steroid feedback, and neuron-glia communication. These inputs ultimately converge on the GnRH pulse-generating system and shape its excitability, rhythmicity, and neuroendocrine output (27–29).

Among the central neuropeptides involved in pubertal regulation, kisspeptin, encoded by the KISS1 gene, is a major upstream regulator of GnRH neuronal activation and pubertal onset (30). In humans, hypothalamic kisspeptin neurons are located primarily in the arcuate nucleus (ARC)/infundibular nucleus and the preoptic area (POA) (31). KNDy neurons in the ARC coexpress kisspeptin, neurokinin B (NKB), and dynorphin (Dyn) and are considered an essential component of, or an upstream regulatory unit for, the GnRH pulse generator. These neurons also participate in the negative feedback regulation of sex steroids (27, 32). The synchronized activity of KNDy neurons and their intrinsic kisspeptin, NKB, and Dyn signaling collectively contribute to the generation of pulsatile GnRH/luteinizing hormone (LH) rhythms (33). By contrast, POA kisspeptin neurons primarily mediate estradiol-positive feedback and regulate the preovulatory GnRH/LH surge. They may also modulate GnRH neuronal excitability through coupling with γ-aminobutyric acid (GABA)ergic or glutamatergic signaling (34). GnRH neurons express the kisspeptin receptor KISS1R/GPR54. Binding of kisspeptin to this receptor activates downstream signaling pathways, promotes GnRH release, and regulates HPG axis function (16, 27).

From an anatomical perspective, the hypothalamic POA, ARC, and median eminence (ME) constitute three key nodes in the network governing pubertal onset. The POA contains GnRH neuronal cell bodies and their upstream synaptic inputs and is therefore an important site for regulating GnRH neuronal excitability. The ARC integrates reproductive and metabolic signals through KNDy, pro-opiomelanocortin (POMC), and neuropeptide Y/agouti-related peptide (NPY/AgRP) neurons. The ME serves as the neuroendocrine interface at which GnRH nerve terminals release GnRH into the hypophyseal portal system. The spatial separation between GnRH neuronal cell bodies and nerve terminals permits regulation at two levels: upstream neuronal networks within regions such as the POA and ARC and the local neuro-glial-vascular release interface in the ME (35, 36). In the ME, GnRH nerve terminals, tanycytes, astrocytes, microglia, vascular endothelial cells, and portal capillaries collectively form a dynamic release interface. Structural plasticity at this interface can alter the proximity of GnRH terminals to portal vessels and thereby influence the efficiency of GnRH entry into the hypophyseal portal system (35, 37).

GnRH neurons and kisspeptin/KNDy neuronal networks are regulated not only by classical neural circuits but also by the hypothalamic neuroimmune microenvironment, which encompasses glial cells, vascular-associated cells, and metabolic and nutritional signals (38). Developmental maturation of the GnRH system is accompanied by dynamic remodeling of neuronal morphology, synaptic organization, glial interactions, and the neurovascular interface, thereby supporting the establishment of GnRH secretory patterns around puberty (39). In the POA, astrocytes lie in close apposition to GnRH neuronal cell bodies and may regulate GnRH-related networks through the release of gliotransmitters. Prostaglandin E2 (PGE2), an important glia-derived mediator of GnRH neurosecretion, can be released by astrocytes, microglia, and tanycytes and contributes to the regulation of GnRH neuronal activity and neurosecretion (40). Astrocytes are also closely associated with KNDy neurons in the ARC and may regulate the excitability and rhythmicity of the GnRH network by coordinating the firing of KNDy neuronal clusters (41). In the ME, tanycytic end-feet dynamically ensheath GnRH nerve terminals and contribute to the regulation of the blood–hypothalamus/cerebrospinal fluid barrier, the sensing of energy-related signals, molecular transport, and the gating of GnRH terminal release (36). Microglia are the resident immune cells of the central nervous system. Beyond their roles in developmental synaptic pruning, tissue homeostasis, and injury responses, they may also contribute to HPG axis regulation. Their regulation of the GnRH system appears to be region specific. In the ME, normal microglial contact with and engulfment of GnRH nerve terminals may be maintained through receptor activator of nuclear factor-κB (RANK) signaling, thereby contributing to the generation and amplification of pubertal GnRH pulses (20). In the POA, a subset of cyclooxygenase-1 (COX-1)-expressing microglia lies in close spatial proximity to GnRH neurons. This distribution suggests that microglia may regulate GnRH neuronal activity through the synthesis and release of prostaglandins and may act synergistically with astrocytes (42).

**Figure 1:**
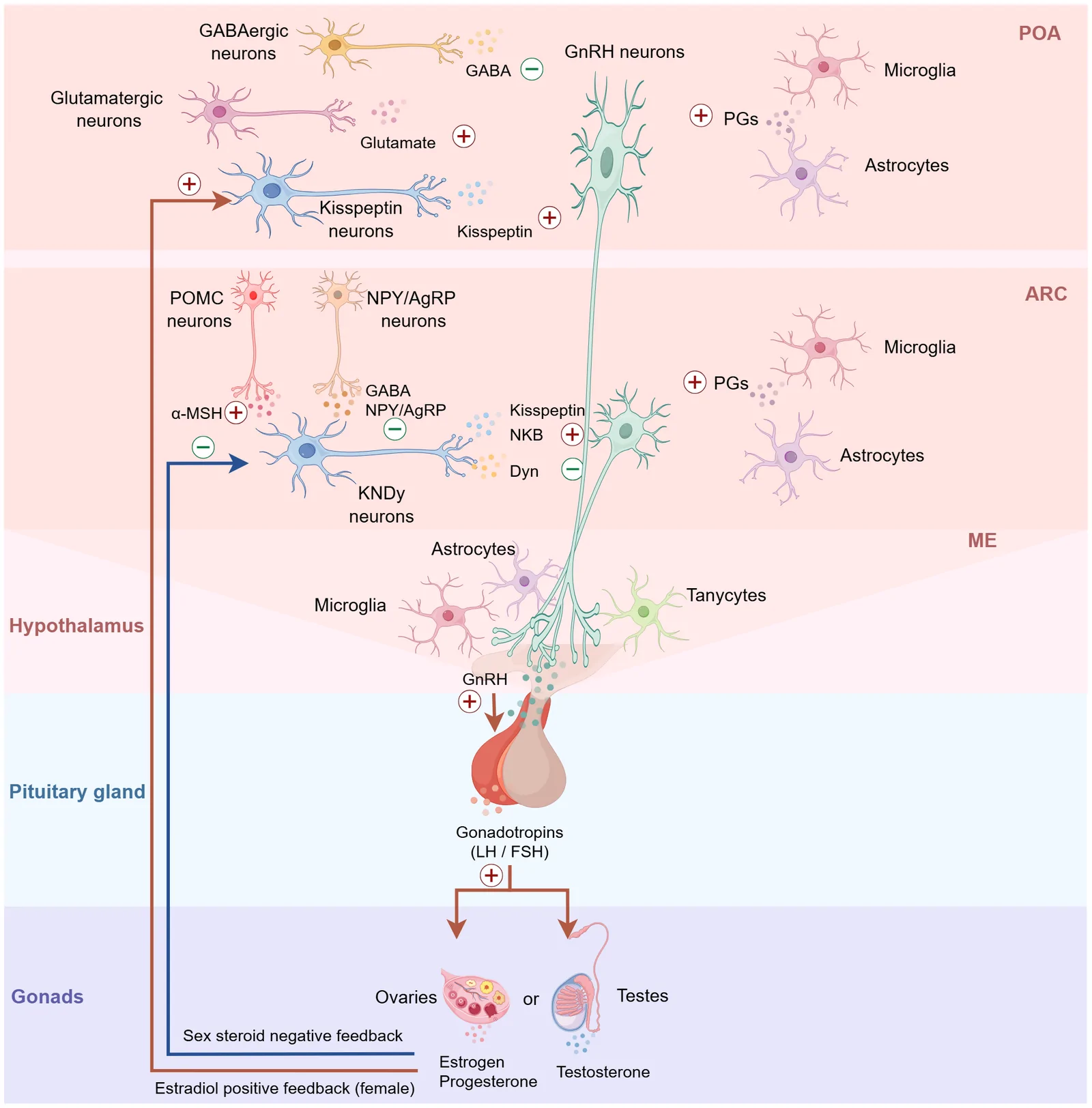
Hypothalamic neuroendocrine and glial–immune regulatory networks involved in pubertal onset. In the POA, GnRH neuronal cell bodies and kisspeptin-related upstream inputs primarily participate in the regulation of GnRH neuronal excitability, estradiol-positive feedback, and GnRH/LH surge-like release. In the ARC, KNDy neurons and metabolic-sensing neurons, including POMC and NPY/AgRP neurons, jointly integrate reproductive and metabolic signals and participate in GnRH pulse regulation. In the ME, GnRH nerve terminals, tanycytes, astrocytes, microglia, and portal vessels collectively form the neuro-glial-vascular interface for GnRH release. Within the POA and ARC, astrocytes and microglia may further contribute to the regulation of kisspeptin-GnRH network excitability and HPG axis activation through mechanisms such as gliotransmitter release, synaptic/network remodeling, immune surveillance, and inflammatory signal sensing. AgRP, agouti-related peptide; ARC, arcuate nucleus; Dyn, dynorphin; FSH, follicle-stimulating hormone; GABA, γ-aminobutyric acid; GnRH, gonadotropin-releasing hormone; HPG, hypothalamic–pituitary–gonadal axis; KNDy, kisspeptin/neurokinin B/dynorphin-expressing neurons; LH, luteinizing hormone; ME, median eminence; NKB, neurokinin B; NPY, neuropeptide Y; POA, preoptic area; PGs, prostaglandins; POMC, pro-opiomelanocortin; α-MSH, α-melanocyte-stimulating hormone. Image created by Figdraw.

Taken together, pubertal onset should be understood not as the isolated, autonomous activation of GnRH neurons but as the outcome of coordinated maturation of the POA-ARC-ME neuroendocrine network and the hypothalamic glial-immune microenvironment. GnRH neuronal cell bodies and kisspeptin-related upstream inputs in the POA; KNDy neurons and metabolic-sensing POMC and NPY/AgRP neurons in the ARC; and GnRH nerve terminals, tanycytes, astrocytes, microglia, and portal vessels in the ME collectively constitute the core spatial framework of pubertal onset. This framework provides a structural basis for understanding how metabolic and inflammatory signals regulate the kisspeptin-GnRH network within specific hypothalamic regions and thereby influence the activation threshold of the HPG axis (**Figure 1**).

### 2.2 Metabolic cues in pubertal timing

Nutritional status and metabolic signals are important regulators of pubertal timing (26). Chronic energy deficiency, as occurs with undernutrition or anorexia, is commonly associated with delayed puberty. Conversely, excess energy storage, particularly in the context of overweight and obesity, is generally associated with earlier pubertal onset (43, 44). Peripheral metabolic hormones, central neurotransmitters, and neuropeptide networks can convey information about energy reserves and nutritional status to the regulatory circuitry of the HPG axis, particularly GnRH neurons and their upstream regulatory neurons (45).

The hypothalamic ARC is a major site of integration for metabolic and reproductive signals. It contains melanocortin system-associated neurons that regulate food intake and energy expenditure, including NPY/AgRP and POMC neurons, as well as kisspeptin/KNDy neuronal networks involved in regulating GnRH pulsatility (46). NPY/AgRP and POMC neurons may influence kisspeptin/KNDy and GnRH-related networks through GABA, NPY/AgRP, α-melanocyte-stimulating hormone (α-MSH), and melanocortin-related signaling, thereby conveying changes in energy status to the reproductive axis. In general, the NPY/AgRP pathway is more commonly associated with reproductive suppression during energy deficiency, whereas the POMC/α-MSH pathway may provide proreproductive signals when energy is sufficient. However, these effects are jointly modulated by developmental stage, sex, the sex steroid milieu, and central metabolic sensitivity (38). As an important component of the GnRH pulse-regulating network in the ARC, KNDy neurons may integrate sex steroid feedback, metabolic hormones, and local signals of nutritional status. They may regulate kisspeptin-GnRH network activity through intracellular energy-sensing pathways, including mechanistic target of rapamycin (mTOR), AMP-activated protein kinase (AMPK), and sirtuin 1 (SIRT1) (22, 46).

Among peripheral metabolic signals, leptin is a representative factor linking adipose energy stores, body weight homeostasis, pubertal onset, and reproductive function (26, 45). Previous studies suggest that leptin does not directly trigger pubertal onset but instead acts as a permissive metabolic signal that enables pubertal development. A threshold level of leptin signaling may be required for reproductive maturation, whereas elevated leptin levels alone are insufficient to initiate puberty (44, 47, 48). Other metabolic hormones, including insulin and ghrelin, may also regulate the metabolic-reproductive axis through GnRH neurons or their upstream networks (6, 23).

Importantly, metabolic signals do not act exclusively through neurons; they may also regulate pubertal onset indirectly through hypothalamic glial cells and local barrier structures. The ARC-ME region lies adjacent to the third ventricle and hypophyseal portal system and contains specialized vascular and barrier structures that allow circulating metabolic signals to access or influence hypothalamic metabolic-sensing networks. Tanycytes can sense and transport energy-related signals such as glucose and leptin and may modulate hypothalamic neuronal responses to the peripheral metabolic environment by regulating the barrier properties of the ARC-ME interface. Astrocytes may influence GnRH/kisspeptin-related networks through metabolic support, gliotransmitter release, and local paracrine signaling. Microglia can sense metabolic stress and inflammatory signals and may affect neuronal excitability and synaptic remodeling by altering the local immune microenvironment (19, 49).

The effects of metabolic signals on pubertal onset therefore cannot be reduced to a simple linear, facilitatory relationship. Instead, they are shaped by multiple interacting regulatory layers, including central metabolic sensitivity (9, 46), the signal integration capacity of the ARC-ME region, hypothalamic inflammatory status (24, 50), peripheral inflammatory burden (51, 52), and the integration of neuropeptide and neurotransmitter pathways involving KNDy, POMC, NPY/AgRP, and GABA signaling (44, 53). This complexity suggests that obesity-associated earlier pubertal onset cannot be explained solely by increased energy reserves. Greater attention should therefore be directed toward the immunoinflammatory mechanisms underlying metabolic signal dysregulation.

### 2.3 From metabolic excess to metaflammation

Metaflammation is a chronic, low-grade, systemic immunoinflammatory state induced by overnutrition and metabolic overload (54–56). This concept provides a framework for understanding how obesity-related metabolic signals may shift from permissive regulators of puberty to drivers of pubertal advancement. Children with overweight or obesity frequently exhibit elevated levels of inflammatory markers, including C-reactive protein (CRP), interleukin-6 (IL-6), IL-1β, and tumor necrosis factor-α (TNF-α) (57, 58). These inflammatory mediators may affect multiple organs involved in metabolic regulation, including adipose tissue, the gut, liver, pancreas, and central nervous system, thereby further disrupting energy homeostasis (9, 59, 60).

In obesity, adipose tissue dysfunction can promote macrophage infiltration and phenotypic remodeling toward a “metabolically activated” state. This phenotype is distinct from classical M1 polarization and is characterized by the upregulation of proinflammatory mediators such as IL-1β and TNF-α (61). Concurrently, obesity-associated changes in the composition and metabolic function of the gut microbiota can compromise intestinal barrier integrity, facilitate the translocation of microbe-associated molecular patterns such as LPS into the circulation, and further activate peripheral and central immunoinflammatory responses (62, 63). Obesity is therefore not merely characterized by increased energy storage but represents a systemic immunometabolic state involving adipose tissue immune remodeling, gut–immune axis dysregulation, elevated circulating inflammatory mediators, and increased susceptibility to central inflammation.

When peripheral metaflammatory signals reach the hypothalamus, they may alter glial responses, neurovascular barrier function, and the activity of metabolic-sensing neurons in the ARC-ME region. These changes may convert normally “permissive” metabolic signals into pathological neuroimmune inputs that promote premature HPG axis activation. From the perspective of hypothalamic spatial organization, metaflammation may primarily act at three levels. First, within the ARC, inflammatory signals may disrupt the integration of nutritional and hormonal signals by POMC, NPY/AgRP, and kisspeptin/KNDy neurons, thereby altering the balance between metabolic and reproductive signaling. Second, within the ME, inflammation and metabolic stress may perturb neuro-glial-vascular interactions among tanycytes, astrocytes, microvascular endothelial cells, and GnRH nerve terminals, thereby altering the gating of GnRH release. Third, within the POA and its associated input networks, the local inflammatory state and glial responses may influence GnRH neuronal excitability and upstream synaptic regulation.

Childhood obesity should therefore be viewed not merely as an energy imbalance caused by overnutrition but as an immunometabolic disorder in which immune activation, metabolic dysregulation, and maladaptive neuroendocrine responses are intertwined (56, 62, 64). Compared with the traditional energy reserve model, this framework provides a more integrated basis for understanding how metabolic disturbances may alter pubertal timing through dysregulation of the immune microenvironment.

## 3 Clinical evidence linking childhood metabolic disorders to earlier pubertal onset

**Figure 2:**
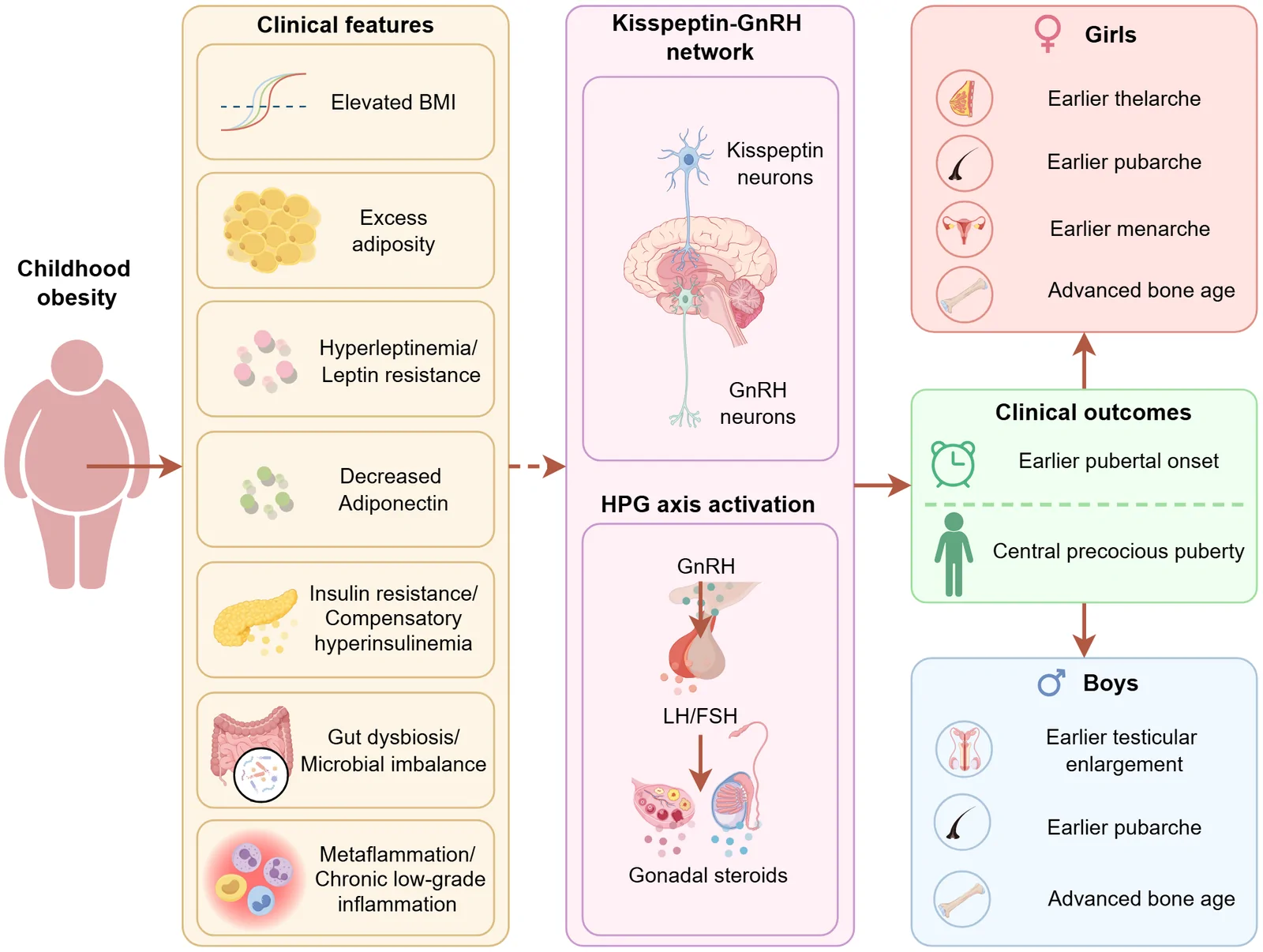
Clinical evidence framework linking childhood obesity-associated metabolic dysregulation to earlier pubertal onset. Childhood obesity represents an important clinical context linking metabolic dysregulation to altered pubertal timing and is often accompanied by clinical features such as hyperleptinemia/leptin resistance, reduced adiponectin, insulin resistance/compensatory hyperinsulinemia, gut dysbiosis, and metaflammation. These obesity-associated metabolic–endocrine and microecological abnormalities may influence the activation threshold of the hypothalamic kisspeptin–GnRH network and HPG axis, thereby contributing to earlier pubertal onset or the development of central precocious puberty. Evidence in girls is relatively consistent, mainly involving earlier thelarche, earlier pubarche, earlier menarche, and advanced bone age; evidence in boys remains more heterogeneous and mainly involves earlier testicular enlargement, earlier pubarche, and advanced bone age. BMI, body mass index; GnRH, gonadotropin-releasing hormone; HPG, hypothalamic-pituitary-gonadal axis. Image created by Figdraw.

Childhood obesity provides an important clinical context linking metabolic dysregulation to earlier pubertal onset. Obesity is characterized not only by elevated BMI and excess adiposity but also by systemic metabolic and endocrine abnormalities, including adipokine dysregulation, such as elevated leptin and reduced adiponectin; insulin resistance, hyperinsulinemia, and leptin resistance; and metaflammation. Gut dysbiosis may further exacerbate this inflammatory state. These peripheral immunometabolic disturbances may contribute to earlier pubertal onset and the development of CPP by modulating the hypothalamic kisspeptin-GnRH network and advancing HPG axis activation. However, this association varies by sex and across individuals. This section reviews clinical evidence linking obesity-associated metabolic abnormalities to earlier pubertal development (**Figure 2**).

### 3.1 Sex-specific differences in the association between obesity and pubertal timing

Among the available clinical and epidemiological evidence, the association between obesity and earlier pubertal onset is most consistently observed in girls. Clinical studies have shown that higher childhood BMI, greater adiposity, and accelerated growth trajectories are associated with a younger age at pubertal onset, although these associations vary by sex and age (65, 66). Higher BMI is associated with earlier thelarche, pubarche, and menarche in girls (67, 68). A large multiethnic cohort study involving 129,824 children further showed that childhood obesity was associated with earlier breast development, pubic hair development, and menarche in girls (69). A systematic review and meta-analysis similarly found that girls with obesity experienced menarche earlier than girls with normal weight (70). Children with overweight or obesity are also more likely to exhibit advanced bone age, with both the frequency and magnitude of bone-age advancement being greater in girls (71). Collectively, these findings identify obesity as an important clinical factor associated with advanced pubertal timing in girls. However, this association is unlikely to reflect BMI alone and probably involves a combination of metabolic abnormalities, adipokine dysregulation, inflammation, and altered neuroendocrine signaling (4, 21, 72).

Compared with that in girls, the association between obesity and pubertal timing in boys is more complex. Earlier studies produced inconsistent findings: some suggested that obesity may be associated with delayed puberty in boys, whereas others found no significant association (70, 73). More recent large-scale studies and systematic reviews, however, suggest that overweight and obesity may also be associated with earlier pubertal development in boys (69, 74, 75). The same large cohort study reported an increased risk of earlier testicular development and pubic hair development among boys with severe obesity, with evidence of a dose-response relationship (69). Another large-scale study found that overweight was associated with earlier spermarche in boys (68). Cross-sectional evidence likewise indicates a higher prevalence of advanced bone age among boys with overweight or obesity (71). A systematic review and meta-analysis further identified significant associations between overweight or obesity and pubertal development in boys, primarily reflected by earlier testicular enlargement and pubic hair development (75). Overall, the evidence in boys remains heterogeneous and may be influenced by differences in pubertal assessment criteria, BMI stratification, racial or ethnic background, and study design. Further longitudinal studies are therefore required.

### 3.2 Adipokine imbalance and metabolic hormone abnormalities as clinical indicators of obesity-associated earlier pubertal onset

Childhood obesity is frequently accompanied by adipokine dysregulation and metabolic hormone abnormalities, including hyperleptinemia, leptin resistance, reduced adiponectin levels, insulin resistance, and compensatory hyperinsulinemia. These changes not only reflect the endocrine consequences of adipose tissue expansion but also suggest that the immunometabolic state associated with obesity may participate in regulating HPG axis function and pubertal timing (76, 77). Clinical studies have shown that girls with CPP have higher serum leptin levels and lower adiponectin levels than healthy controls (78, 79). Systematic reviews and meta-analyses have similarly identified altered adipokine profiles in girls with CPP, characterized by elevated leptin and reduced adiponectin levels relative to those in healthy girls (80).

Insulin resistance and compensatory hyperinsulinemia provide additional metabolic clues linking childhood obesity to earlier pubertal onset. Among girls with CPP, those with higher BMI have greater bone age, more advanced bone-age progression, higher insulin levels, and higher indices of insulin resistance. Moreover, insulin resistance is positively correlated with both bone-age advancement and BMI (81). Another cohort study identified insulin resistance as an independent risk factor for earlier pubertal onset in children and suggested that it may partially mediate the association between environmental exposures and advanced pubertal timing (82). These findings indicate that obesity-associated earlier pubertal onset should not be attributed solely to elevated BMI. Instead, it should be comprehensively evaluated alongside metabolic and endocrine phenotypes, including leptin and adiponectin levels, insulin resistance, bone-age advancement, and sex hormone levels.

### 3.3 Gut dysbiosis as a clinical microecological indicator of obesity-associated earlier pubertal onset

Gut dysbiosis represents a potential microecological link between metabolic dysregulation associated with childhood obesity and earlier pubertal development or precocious puberty. Clinical studies have identified alterations in gut microbiota composition and diversity among girls with obesity and those with obesity-associated precocious puberty. Shared microbial features between obesity and CPP suggest that the gut microbiota may be involved in obesity-associated precocious puberty (83, 84). Other studies have reported altered profiles of gut microbiota-derived metabolites in children with CPP, particularly reduced levels of short-chain fatty acids (SCFAs), such as butyrate and propionate (85). Children with obesity-associated precocious puberty exhibit gut microbial profiles distinct from those of children with obesity alone or precocious puberty alone. These differences may reflect a specific immunometabolic phenotype associated with obesity-related advancement of pubertal onset (86). Collectively, these findings provide clinical clues to the microecological mechanisms that may underlie obesity-associated earlier pubertal onset.

Based on the available evidence, obesity-associated earlier pubertal onset or CPP cannot be explained by BMI alone. Instead, it may represent a clinical immunometabolic phenotype characterized by coordinated alterations across multiple dimensions, including physical development, adipokines, metabolic hormones, bone-age advancement, and the gut microbiota. **Table 1** summarizes the relevant candidate biomarkers and supporting clinical evidence.

**Table 1:** Candidate biomarkers and clinical evidence for obesity-associated earlier pubertal onset and CPP in children.

Biomarker category	Candidate biomarkers	Associated clinical outcomes	Study population and evidence type	Main clinical findings and limitations	References
Anthropometric indices	BMI, BMI z-score/BMI SDS, BMI velocity, waist circumference, adiposity and growth trajectories	Earlier pubertal onset; CPP/ICPP; earlier thelarche, pubarche and menarche in girls; earlier testicular development, pubarche and spermarche in boys	Girls with ICPP; population-based and multiethnic pediatric cohorts; longitudinal and cross-sectional studies; systematic reviews/meta-analyses	Higher childhood adiposity and sustained prepubertal BMI gain are associated with earlier pubertal onset and increased CPP risk, particularly in girls, whereas evidence in boys remains heterogeneous. These indices aid monitoring and risk stratification but lack diagnostic specificity.	(4, 5, 21, 65–70, 72, 73, 75)
Skeletal maturation	Bone age, bone age advancement, bone age–chronological age difference	Advanced skeletal maturation; earlier pubertal development; CPP	Overweight/obese children; girls with CPP, non-CPP and healthy controls; cross-sectional and retrospective clinical studies	Overweight and obesity are associated with advanced bone age; in girls with CPP, greater advancement is also associated with higher BMI and insulin resistance. Bone age is clinically useful but nonspecific.	(71, 81)
Neuroendocrine and epigenetic biomarkers	Kisspeptin, MKRN3, MBD3	CPP; premature thelarche; HPG-axis activation	Girls with CPP, premature thelarche, or healthy prepubertal controls; observational and diagnostic studies	Increased kisspeptin, reduced MKRN3 and altered MBD3 may reflect upstream HPG-axis regulation. Diagnostic performance remains insufficient for use as standalone markers.	(87–89)
Adipokines	Leptin, total adiponectin, high-molecular-weight adiponectin, omentin-1, chemerin	CPP/ICPP; metabolic and immune–endocrine phenotype	Girls with CPP/ICPP and healthy controls; observational studies; systematic review/meta-analysis	CPP is generally associated with higher leptin and lower total adiponectin; omentin-1 and chemerin may provide additional immunometabolic information. Results are strongly influenced by BMI, adiposity and pubertal stage.	(78–80, 90)
Inflammatory mediators	IL-6, CRP, TNF-α	Pubertal timing; CPP inflammatory phenotype; intervention response	General pubertal cohorts and girls with CPP; observational and exercise-intervention studies	Systemic inflammatory markers may be associated with pubertal timing and change alongside CPP-related outcomes after exercise. Associations may weaken after adjustment for adiposity and are not CPP-specific.	(52, 91)
Immune cell-related indicators	White blood cell count, neutrophils, lymphocytes, neutrophil-to-lymphocyte ratio (NLR)	Obesity-associated CPP; systemic low-grade inflammation; intervention response	Healthy controls, children with obesity, CPP or obesity-associated precocious puberty; cross-sectional and exercise-intervention studies	Obesity-associated CPP is accompanied by higher WBC and NLR, while exercise-related decreases in WBC and neutrophils may parallel improvements in pubertal indices. These indicators are inexpensive but susceptible to infection, stress, adiposity, and other confounders.	(91, 92)
Metabolic and growth-axis biomarkers	Fasting insulin, HOMA-IR, IGF-1, IGFBP-3, IGF-1 SDS, IGFBP-3 SDS	Earlier pubertal onset; CPP; advanced bone age	Girls with CPP or obesity-associated CPP and pediatric cohorts; longitudinal, retrospective, cross-sectional, and predictive-model studies	Insulin resistance is associated with accelerated pubertal onset and bone-age advancement, whereas IGF-1 and IGFBP-3 may improve CPP prediction when combined with age and basal gonadotropins. These markers are nonspecific and require age-, sex-, and assay-specific interpretation.	(78, 81, 82, 92–94)
Gut microbiota	Microbial composition and diversity; selected taxa and genera, including Dialister, Bacteroides, Bifidobacterium, Anaerostipes, Sellimonas and Oscillibacter	CPP; obesity-associated precocious puberty; HPG-axis-related outcomes; risk stratification	Children with CPP, obesity-associated precocious puberty or obesity with precocious puberty; clinical studies; systematic review/meta-analysis; Mendelian randomization	CPP and obesity-associated precocious puberty show alterations in microbial composition and candidate taxa, some of which correlate with BMI, bone age and gonadotropins. Results are influenced by diet, age, BMI, region and analytical methods.	(83–86, 95)
Microbial metabolites and functions	SCFAs, particularly butyrate and propionate; NO synthesis-related functions; steroid and neuroactive metabolites	CPP; obesity-associated CPP; noninvasive risk stratification	CPP and overweight/obese children; microbiome–metabolomics studies; systematic review/meta-analysis	CPP is associated with altered microbial metabolic functions, including reduced SCFAs and changes in NO-, steroid- and neurotransmitter-related metabolism. Findings remain exploratory and require independent validation.	(84, 85, 96)
Angiogenic biomarkers	VEGF-A	CPP; Tanner-stage progression; advanced bone age and skeletal maturation	Girls across pubertal stages and girls with precocious puberty; cross-sectional clinical study	VEGF-A is elevated in girls with CPP and is associated with Tanner stage, estradiol and bone age advancement, but its specificity and incremental diagnostic value remain uncertain.	(97)
Composite indicators and multimarker models	Combinations of basal LH/FSH, estradiol, kisspeptin, MKRN3, vitamin D, IGF-1/IGFBP-3, BMI, bone age, immune–metabolic indicators, ultrasound parameters, and microbiome–metabolomics features	CPP; progressive CPP; obesity-associated CPP	Children undergoing evaluation for CPP; retrospective diagnostic studies, predictive-model studies, cross-sectional studies, and multi-omics analyses	Multimarker models generally show better classification performance than individual biomarkers and may better reflect CPP heterogeneity. Most models still lack multicenter external validation, assay standardization, and longitudinal outcome assessment.	(87, 88, 92–94, 96)

BMI, body mass index; CPP, central precocious puberty; CRP, C-reactive protein; FSH, follicle-stimulating hormone; HOMA-IR, homeostatic model assessment of insulin resistance; HPG, hypothalamic–pituitary–gonadal; ICPP, idiopathic central precocious puberty; IGF-1, insulin-like growth factor 1; IGFBP-3, insulin-like growth factor-binding protein 3; IL-6, interleukin-6; LH, luteinizing hormone; MBD3, methyl-CpG-binding domain protein 3; MKRN3, makorin ring finger protein 3; NLR, neutrophil-to-lymphocyte ratio; NO, nitric oxide; SCFAs, short-chain fatty acids; SDS, standard deviation score; TNF-α, tumor necrosis factor alpha; VEGF-A, vascular endothelial growth factor A; WBC, white blood cell count.

## 4 Sources of peripheral metaflammation and immunometabolic signals in childhood obesity

**Figure 3:**
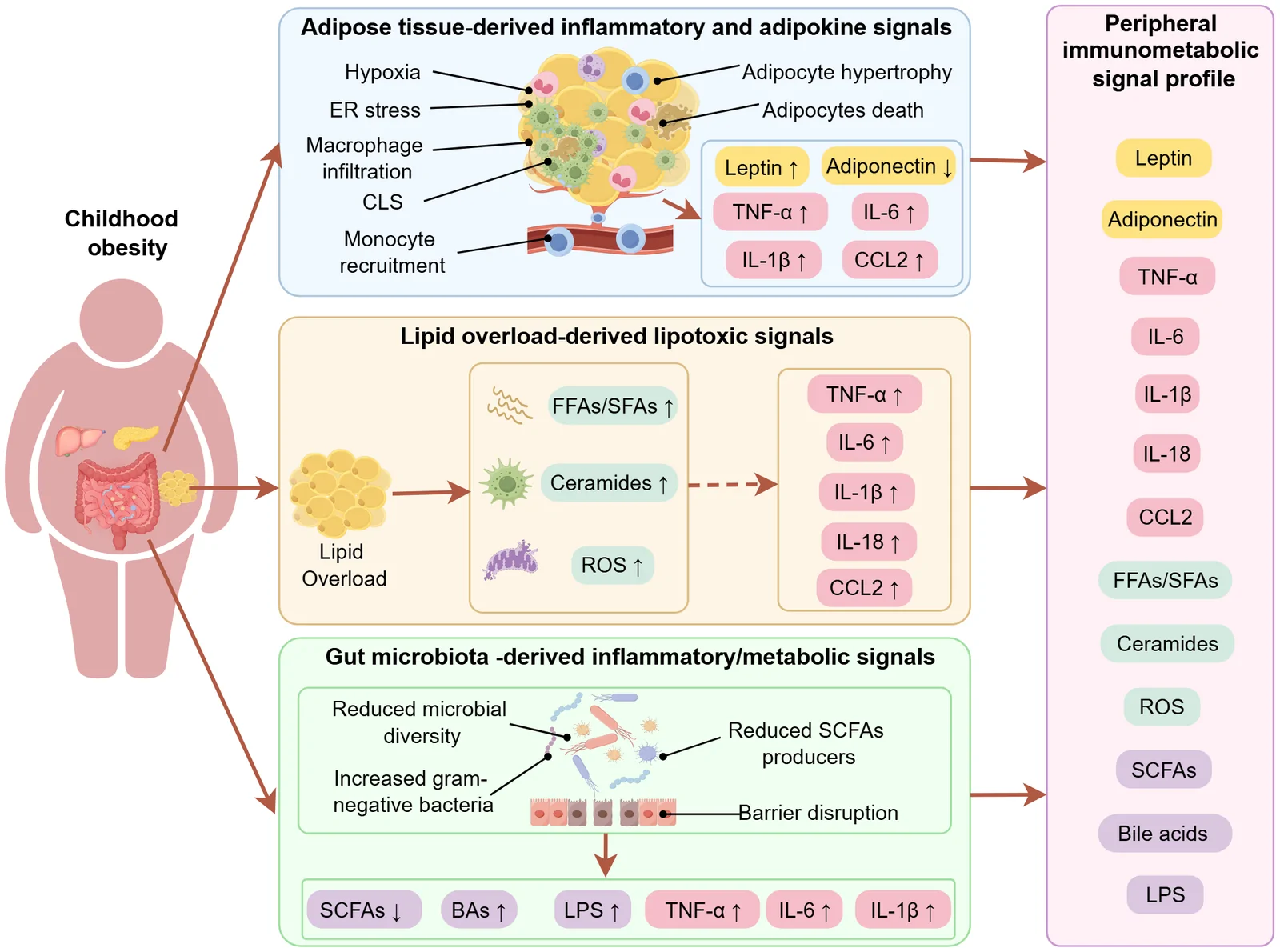
Sources of peripheral metaflammation and immunometabolic signals in childhood obesity. Childhood obesity induces systemic low-grade inflammation mainly through three peripheral sources: adipose tissue inflammation, lipid overload, and gut dysbiosis. Adipose tissue inflammation may promote adipokine imbalance and the release of proinflammatory cytokines. Lipid overload can generate lipotoxic signals, including free fatty acids/saturated fatty acids, ceramides, and reactive oxygen species, and promote the release of inflammatory mediators. Gut dysbiosis may lead to reduced microbial diversity, intestinal barrier dysfunction, and LPS translocation into the circulation, accompanied by alterations in SCFAs, bile acids, and other microbial metabolites. Together, these signals constitute the peripheral immunometabolic signal profile in childhood obesity. BAs, bile acids; CLS, crown-like structures; ER stress, endoplasmic reticulum stress; FFAs, free fatty acids; IL, interleukin; LPS, lipopolysaccharide; MCP-1/CCL2, monocyte chemoattractant protein-1/C-C motif chemokine ligand 2; ROS, reactive oxygen species; SCFAs, short-chain fatty acids; SFAs, saturated fatty acids; TNF-α, tumor necrosis factor-α. Image created by Figdraw.

Childhood obesity is not simply an increase in adipose mass but a systemic immunometabolic state characterized by coordinated alterations in adipose tissue, circulating lipids, and the intestinal immune environment. Adipose tissue expansion, lipid overload, and impaired intestinal barrier function can generate adipokines and cytokines, lipid-derived danger signals, and gut-derived inflammatory and microbial metabolic signals, respectively. Together, these constitute three major sources of peripheral metaflammation. These peripheral immunometabolic signals may act as circulating inputs to the hypothalamic metabolic-reproductive network, thereby providing a peripheral signaling basis for subsequent modulation of the activation threshold of the kisspeptin-GnRH/HPG axis (**Figure 3**).

### 4.1 Adipose tissue-derived inflammatory and adipokine signals

Adipose tissue is not only an energy-storage depot but also an important endocrine and immunometabolic organ that secretes diverse adipokines and cytokines involved in systemic energy homeostasis, insulin sensitivity, and immune regulation (98–100). Under physiological conditions, adipose tissue is relatively insulin sensitive, exhibits low lipolytic activity, and secretes anti-inflammatory adipokines such as adiponectin. In obesity, however, adipose tissue undergoes pathological remodeling characterized by adipocyte hypertrophy, inadequate vascular adaptation, hypoxia, cell death, and increased macrophage infiltration. Its secretory profile shifts toward a proinflammatory phenotype, with elevated leptin, reduced adiponectin, and increased production of proinflammatory mediators such as IL-6, IL-1β, TNF-α, and MCP-1/CCL2 (101–103). This transition is closely associated with macrophage infiltration. MCP-1/CCL2 secreted by adipocytes and cells within the stromal vascular compartment recruits circulating monocytes into adipose tissue. Infiltrating macrophages surround dead adipocytes to form crown-like structures and release additional proinflammatory mediators. These mediators enter the circulation and contribute to chronic systemic low-grade inflammation (104–106). Studies of childhood obesity similarly indicate that children with obesity may exhibit early adipocyte hypertrophy, macrophage infiltration, and crown-like structure formation, which are associated with elevated high-sensitivity C-reactive protein levels and insulin resistance (107). Elevated leptin and reduced adiponectin may contribute to the peripheral metabolic signaling involved in obesity-associated earlier pubertal onset (11).

As adipose tissue continues to expand, local hypoxia activates hypoxia-inducible factor-1α (HIF-1α)-related responses, further exacerbating endoplasmic reticulum stress, cell death, and extracellular matrix remodeling while promoting the release of chemokines and inflammatory mediators (108). In obesity, adipose tissue macrophages not only increase in abundance but also acquire metabolically activated features, including enhanced lipid handling, altered lysosomal function, and upregulated expression of proinflammatory mediators (61). Adipose tissue immune remodeling can therefore translate increased adiposity into sustained cytokine and adipokine signaling. Together with insulin, free fatty acids, and other metabolic stress molecules, these signals constitute the peripheral immunometabolic profile of obesity and may subsequently influence the hypothalamic metabolic-reproductive network (102, 109, 110).

### 4.2 Lipid overload-derived lipotoxic signals

In addition to classical cytokines and adipokines, obesity-associated lipid overload can generate metabolic stress signals, including saturated fatty acids, ceramides, and oxidative stress products, which are important components of peripheral metaflammation (12, 111). Saturated fatty acids, particularly palmitic and stearic acids, are among the major lipid species elevated in obesity (112). Clinical studies have shown that children with obesity have increased serum levels of total fatty acids and multiple saturated fatty acids, which are associated with insulin resistance and adipose tissue inflammation (113, 114). Saturated fatty acids can act as metabolic danger signals in adipocytes, monocytes, and macrophages. Through TLR4/NF-κB-related inflammatory pathways, they promote the expression of proinflammatory mediators such as TNF-α, IL-6, IL-1β, and MCP-1/CCL2, thereby translating overnutrition into sustained peripheral lipotoxic inflammatory signaling (115–117).

Ceramides are important sphingolipid signaling molecules that link nutritional overload, lipid metabolic abnormalities, and low-grade inflammation (118). Saturated fatty acids and LPS can act synergistically to promote ceramide production in macrophages. Ceramide accumulation may further amplify the release of proinflammatory mediators such as IL-1β and TNF-α and contribute to NLRP3 inflammasome activation. This process promotes the maturation and secretion of IL-1β and IL-18, thereby linking lipid overload to innate immune activation and metabolic dysfunction (119–121). Animal studies have also shown that obesity can increase hypothalamic ceramide levels, which are associated with earlier pubertal onset (122). Clinical lipidomic studies have identified abnormalities in ceramides and other serum lipid species among girls with idiopathic CPP (ICPP), suggesting that lipid metabolic remodeling may represent an important peripheral indicator of obesity-associated earlier pubertal onset (123). However, current evidence is derived primarily from animal experiments and cross-sectional studies, and the causal relationships remain to be established (124).

Oxidative stress products are important amplifiers of lipid overload-induced peripheral inflammation. Obesity can increase reactive oxygen species (ROS) production and disrupt redox homeostasis by enhancing nicotinamide adenine dinucleotide phosphate (NADPH) oxidase activity, inducing mitochondrial dysfunction, and reducing antioxidant capacity (125, 126). Excess ROS can activate NF-κB, c-Jun N-terminal kinase (JNK), and the NLRP3 inflammasome, thereby promoting the release of inflammatory mediators such as IL-6, MCP-1, IL-1β, and IL-18 and contributing to obesity-associated insulin resistance and low-grade inflammation (125, 127, 128). Saturated fatty acids, ceramides, and oxidative stress products therefore collectively constitute peripheral lipotoxic inputs in obesity.

### 4.3 Gut microbiota-derived inflammatory and metabolic signals

The gut microbiota is a key regulatory system linking diet, energy metabolism, barrier function, and immunoinflammatory responses (129). In healthy children, the gut microbiota is dominated by major bacterial phyla such as Firmicutes and Bacteroidetes and is shaped by age, diet, and host genetics (130, 131). In obesity, both microbial composition and function are altered. Some studies have reported an increased relative abundance of Firmicutes, a decreased abundance of Bacteroidetes, and an elevated Firmicutes/Bacteroidetes ratio. However, this pattern is not consistent across populations (132–134). Obesity-associated dysbiosis should therefore be understood as a coordinated alteration in microbial composition, metabolic function, intestinal barrier integrity, and host immunoinflammatory responses (135, 136).

Obesity-associated dysbiosis can generate peripheral signals with inflammatory and metabolic regulatory effects by altering energy harvest, SCFA production, bile acid metabolism, and intestinal barrier integrity (135, 137). A reduction in SCFA-producing bacteria may weaken intestinal trophic support and the anti-inflammatory milieu (138, 139), whereas an increased abundance of Gram-negative bacteria, such as Proteobacteria, may increase the LPS burden and exacerbate intestinal barrier disruption and peripheral inflammation (140). LPS is a key microbial molecule that activates innate immunity and promotes metaflammation (141). Obesity and high-fat diets can disrupt the intestinal barrier and alter microbial community structure, thereby increasing circulating LPS levels and inducing “metabolic endotoxemia” (142, 143). Animal studies have shown that high-fat diets can induce metabolic endotoxemia, adipose tissue inflammation, and insulin resistance, whereas modulation of the gut microbiota can reduce LPS levels and inflammation (144). Through signaling pathways such as TLR4/NF-κB, LPS can also induce the release of proinflammatory mediators, including TNF-α, IL-6, and IL-1β, thereby increasing the systemic burden of gut-derived inflammatory signals (145, 146).

In contrast to proinflammatory signals such as LPS, SCFAs and bile acids are important microbiota–host co-metabolic signals. SCFAs are produced primarily through the microbial fermentation of dietary fiber and include acetate, propionate, and butyrate. They contribute to maintaining intestinal barrier integrity, regulating immune cell differentiation, and limiting inflammatory responses (147). Bile acids undergo metabolic transformation by the gut microbiota and participate in energy metabolism, inflammatory regulation, and endocrine signaling (148). Alterations in SCFAs and bile acids in childhood obesity may reflect the combined influences of dietary composition, microbial communities, intestinal absorption, and host metabolic status. These metabolites should therefore be regarded as peripheral indicators of microbial metabolic function rather than being simplistically classified as either proinflammatory or anti-inflammatory factors (149–151).

Taken together, gut dysbiosis may generate two categories of peripheral inputs in childhood obesity: a proinflammatory burden represented by LPS and gut-derived inflammatory mediators, and nutrition-immune regulatory signals represented by SCFAs and bile acids. Current evidence suggests that the gut microbiota and its metabolites may influence HPG axis activity and are associated with earlier pubertal onset or precocious puberty (152). Gut-derived inflammatory and microbial metabolic signals, together with adipose tissue inflammation and lipotoxic signals, may therefore constitute peripheral immunometabolic inputs to the hypothalamic metabolic-reproductive network.

## 5 Potential pathways for the central transmission of peripheral metaflammatory signals

**Figure 4:**
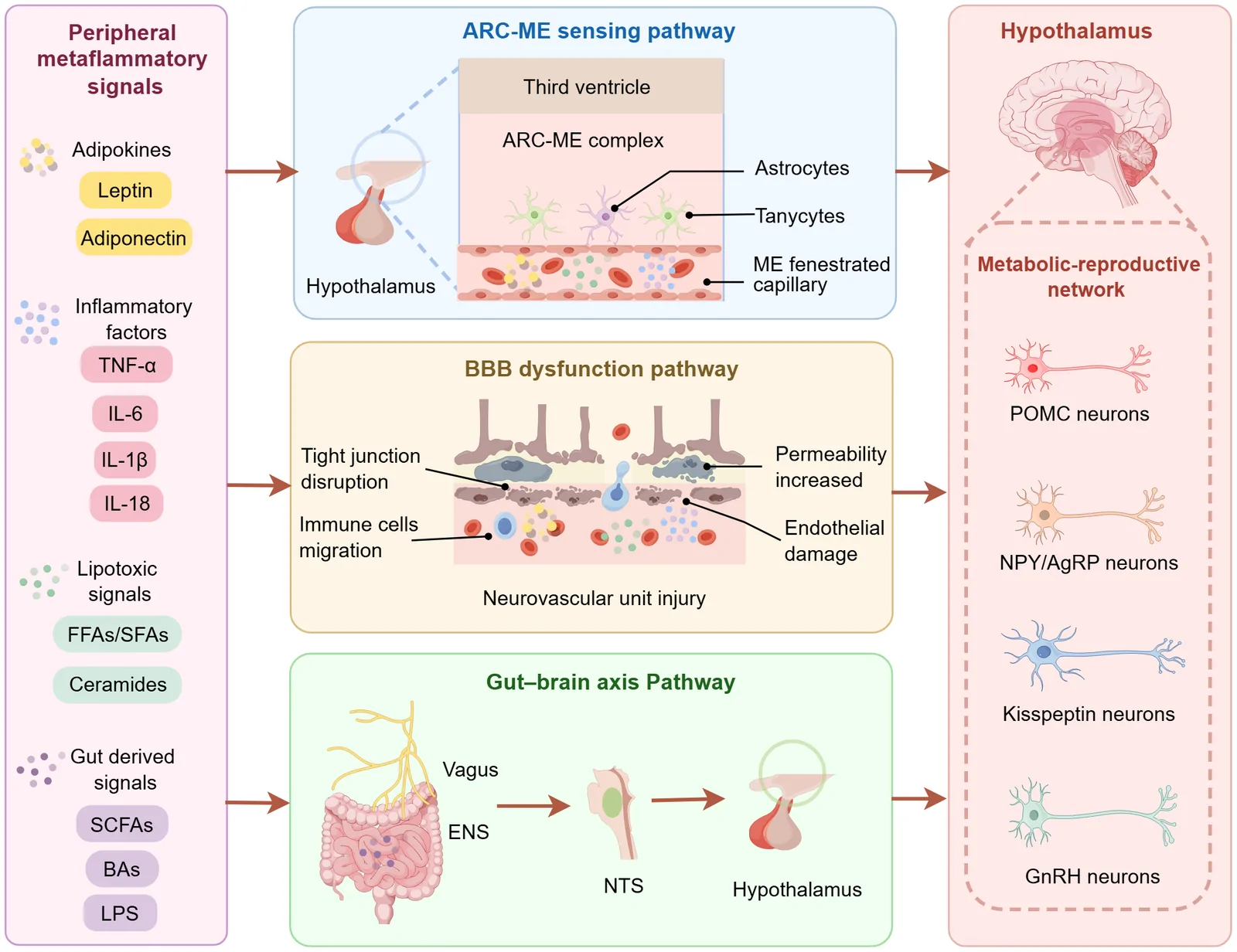
Potential pathways for the transmission of peripheral metaflammatory signals to the hypothalamus. Peripheral metaflammatory signals may be sensed by the hypothalamus through three complementary pathways. First, blood-borne signal sensing mediated by the ARC–ME complex allows circulating inflammatory mediators, adipokines, and lipotoxic signals to be detected by ARC neurons through the fenestrated capillaries characteristic of the ME and the neuro–glial–vascular interface formed by tanycytes, astrocytes, and vascular-associated cells. Second, inflammatory signal amplification mediated by BBB dysfunction may occur when persistent low-grade inflammation, lipid metabolic dysregulation, and endotoxemia impair neurovascular unit integrity, leading to tight junction disruption, endothelial dysfunction, increased permeability, and peripheral immune cell trafficking, thereby enhancing the impact of circulating inflammatory signals on the hypothalamic parenchyma. Third, neuroimmune signal transmission mediated by the gut–brain axis enables gut-derived signals to influence the ARC–ME/BBB interfaces through the circulation or to reach the hypothalamus through vagal afferent pathways relayed by the nucleus tractus solitarius (NTS) in the brainstem. These pathways ultimately converge on the hypothalamic metabolic–reproductive network. AgRP, agouti-related peptide; ARC, arcuate nucleus; BBB, blood-brain barrier; ENS, enteric nervous system; GnRH, gonadotropin-releasing hormone; ME, median eminence; NPY, neuropeptide Y; NTS, nucleus tractus solitarius; POMC, pro-opiomelanocortin. Image created by Figdraw.

Adipokines and cytokines, lipotoxic signals, gut-derived inflammatory products, and microbial metabolites do not remain confined to the periphery but can be sensed by the hypothalamus through multilayered peripheral-central communication mechanisms. The hypothalamus is not only a central regulator of energy homeostasis but also a major integrative site for pubertal onset and HPG axis activation. Accordingly, it is highly sensitive to circulating nutrients, metabolic hormones, inflammatory mediators, and gut-derived signals. The transmission of peripheral metaflammatory signals to the hypothalamus can be conceptualized as three interwoven pathways: blood-borne signal sensing mediated by the ARC-ME complex, amplification of inflammatory signaling through BBB dysfunction, and neuroimmune signal transmission through the gut-brain axis. Together, these pathways integrate obesity-associated metabolic, inflammatory, and gut-derived signals within the hypothalamus, providing a potential mechanistic basis for their subsequent effects on kisspeptin neurons, the GnRH secretory network, and the timing of HPG axis activation (**Figure 4**).

### 5.1 Blood-borne signal sensing mediated by the ARC-ME complex

Peripheral inflammatory mediators, adipokines, lipid metabolites, and gut-derived signals can act on the hypothalamus through the circulation, providing a potential route by which peripheral metaflammation influences central neuroendocrine regulation (9). Unlike most brain regions, certain hypothalamic areas adjacent to the third ventricle possess specialized vascular and barrier structures that enable them to sense blood-borne signals without requiring widespread BBB disruption. These structures form region-specific windows through which peripheral metabolic and inflammatory signals can influence the central nervous system (153).

Circumventricular organs are structurally specialized regions surrounding the ventricular system. They are characterized by relatively high vascular permeability and the capacity to detect circulating signals rapidly (154). Among these structures, the ME is located at the floor of the third ventricle and serves as an interface between the hypothalamic neuroendocrine system and the hypophyseal portal circulation (155). Together with the adjacent ARC, the ME forms the ARC-ME complex, an important functional unit in which metabolic, reproductive, and inflammatory signals converge (156). This region is enriched in neurons involved in energy homeostasis, including POMC and AgRP/NPY neurons, and is functionally connected to kisspeptin neurons and the GnRH secretory network.

Although the microvessels of the ME are fenestrated, the entry of blood-borne signals into the ARC is not governed solely by passive diffusion. Instead, it is regulated by a neuro-glial-vascular interface comprising tanycytes, astrocytes, vascular endothelial cells, and pericytes (37, 157). Tanycytes are specialized ependymoglial cells lining the wall of the third ventricle. They participate in the dynamic regulation of the blood-hypothalamus/cerebrospinal fluid barrier and influence the efficiency with which circulating metabolic signals enter the hypothalamus (158). Under conditions of obesity or overnutrition, circulating free fatty acids, inflammatory mediators, adipokines, and gut-derived LPS may act on local hypothalamic cells through the ARC-ME interface. The ARC-ME complex may therefore serve as an important blood-borne sensing window through which peripheral immunometabolic signals access the hypothalamic metabolic-reproductive network (159). In addition, the axon terminals of GnRH neurons project to the ME, where GnRH is released into the hypophyseal portal system (160), positioning this region at the intersection of peripheral metabolic sensing and reproductive endocrine output.

### 5.2 Inflammatory signal amplification mediated by BBB dysfunction

The BBB is essential for maintaining homeostasis between the peripheral circulation and the central nervous system. It selectively regulates the exchange of ions, cells, and molecules between the vascular lumen and brain parenchyma while restricting the entry of inflammatory mediators and peripheral immune cells into the central compartment (161). The structural and functional integrity of the BBB depends on the neurovascular unit (NVU), which comprises brain microvascular endothelial cells, pericytes, astrocytic end-feet, microglia, the basement membrane, and neurons. Together, these components maintain tight junction integrity, transport functions, vascular stability, and immune barrier properties (162–166).

In obesity and overnutrition, the metabolic overload induced by diets high in fat and sugar, together with systemic low-grade inflammation, can compromise NVU integrity and lead to BBB dysfunction (167). The underlying mechanisms include reduced expression or redistribution of tight junction proteins, endothelial dysfunction, altered transporter expression, increased transcytosis, enhanced oxidative stress, and increased cerebral microvascular permeability (168, 169). At the cellular level, obesity-associated BBB dysfunction involves abnormalities across multiple NVU components. Brain microvascular endothelial cells directly maintain the selective permeability of the BBB, and exposure to a high-fat diet or obesity can disrupt endothelial tight junctions and the transport of metabolic signals (169, 170). Pericytes support barrier homeostasis by maintaining vascular stability and capillary permeability, and their dysfunction can exacerbate BBB leakage (171). Astrocytic end-feet ensheath brain capillaries and maintain BBB integrity through extensive contact with endothelial cells (166). Under metabolic stress, microglia may become activated and release proinflammatory mediators that further disrupt tight junctions and promote the adhesion and migration of peripheral immune cells (172). Obesity-associated BBB dysfunction should therefore be understood as an NVU remodeling process involving endothelial cells, pericytes, astrocytes, microglia, and other cellular components (173).

Concurrently, impaired insulin and leptin signaling, dysregulated lipid metabolism, and gut-derived endotoxemia may act together on the BBB and further weaken its barrier function (9, 174, 175). The BBB is not merely a physical barrier but also an important interface for the central transduction of peripheral inflammatory signals. During short-term or mild inflammation, the BBB may participate in the adaptive relay of peripheral immune signals. As systemic inflammation persists, however, inflammatory cytokines, lipid-derived danger signals, gut-derived LPS, and other inflammatory mediators can disrupt the transport and metabolic functions of cerebral endothelial cells, increase BBB permeability, and potentially facilitate the migration of peripheral immune cells into the central nervous system (161, 176–179). BBB dysfunction may therefore serve as a pathological amplification pathway for the central transmission of peripheral metaflammation. Unlike the region-specific blood-borne sensing mediated by the ARC-ME complex, BBB disruption may allow circulating inflammatory mediators to exert broader effects on the hypothalamic parenchyma, thereby creating conditions that favor hypothalamic neuroimmune responses and alterations in the metabolic-reproductive network.

### 5.3 Neuroimmune signal transmission mediated by the gut-brain axis

In addition to ARC-ME-mediated blood-borne sensing and BBB dysfunction, gut inflammation and microbial signals may influence central metabolic and inflammatory sensing through the gut-brain axis. The microbiota-gut-brain axis is a bidirectional communication network linking the intestine and the central nervous system. It involves the gut microbiota and its metabolites, the intestinal barrier, mucosal immunity, the enteric nervous system (ENS), the vagus nerve, and circulating metabolic mediators (180).

Diets high in fat and sugar can alter gut microbial composition and metabolic function, compromise intestinal barrier integrity, increase the translocation of bacterial products such as LPS into the circulation, and promote systemic low-grade inflammation (181–183). The resulting gut-derived signals, including LPS, proinflammatory mediators, SCFAs, and bile acids, may be transmitted to the central nervous system through three routes. The first is blood-borne input, whereby these signals act through the circulation on hypothalamic sensing interfaces such as the ARC-ME complex or gain greater access to the brain parenchyma when the BBB is impaired. The second route involves local neuroimmune signaling within the ENS (184). The ENS is organized into the myenteric and submucosal plexuses and comprises enteric neurons and enteric glial cells (185). Intrinsic primary afferent neurons can recognize bacterial components such as LPS and metabolic signals such as SCFAs and bile acids through cell-surface pattern-recognition receptors and metabolite sensors. The ENS can also sense inflammation-related signals within the luminal microenvironment indirectly through epithelial sensory cells such as enterochromaffin cells. Through these mechanisms, the ENS regulates intestinal motility, secretion, barrier integrity, and local inflammatory responses; modifies local neural circuit activity; and shapes the intensity and pattern of gut-derived sensory signals transmitted to the central nervous system (186–188). Gut-derived signals may also act on enteric neurons, enteric glial cells, and mucosal immune cells, thereby altering ENS activity and modulating the firing and signal transmission of vagal afferent fibers through enterochromaffin cell-mediated neuroepithelial circuits (189, 190). The third route involves vagus nerve-mediated neural input, whereby gut-derived signals are transmitted through vagal afferent pathways to the nucleus tractus solitarius (NTS) in the brainstem and subsequently influence hypothalamic metabolic and neuroendocrine functions (191–193).

The vagus nerve provides a rapid neural route for gut-brain communication that is distinct from blood-borne input. Its afferent fibers can directly sense certain gut-derived signals and may also receive luminal information indirectly through enteroendocrine cells, immune cells, and the ENS (192, 194). Vagal afferent neurons can express multiple receptors involved in metabolic and inflammatory sensing, including FFAR2/FFAR3, TGR5, TLR4, and cytokine receptors such as IL-1 and TNF receptors. These receptors enable vagal afferents to respond to SCFAs, bile acids, LPS, and inflammatory mediators (195, 196). In indirect transmission, enteroendocrine cells sense nutrients and microbial metabolites and release signaling molecules such as cholecystokinin (CCK), glucagon-like peptide-1 (GLP-1), peptide YY (PYY), and 5-HT. These molecules act on vagal afferent terminals through receptors such as CCK1R, GLP-1R, NPY/PYY-related receptors, and 5-HT3R (197, 198). Mediators released by mucosal immune cells, including IL-1β, IL-6, TNF-α, and prostaglandins, may also modulate vagal sensitivity (199). Vagal efferent pathways can, in turn, regulate intestinal motility, secretion, barrier function, and immune responses and constrain excessive proinflammatory activity through the inflammatory reflex, thereby forming a bidirectional central-peripheral regulatory loop (200).

The gut-brain axis therefore contributes to the central transmission of peripheral metaflammation through two complementary mechanisms: blood-borne input of gut-derived inflammatory products and vagus nerve-mediated neural input. The former primarily reflects the cumulative burden of chronic inflammation, whereas the latter facilitates rapid neural signal transduction. Together, these mechanisms translate diet-induced dysbiosis, intestinal barrier disruption, and peripheral immune activation into neuroimmune-metabolic signals that can be sensed by the hypothalamus.

Taken together, the three pathways of peripheral-central communication are mechanistically interconnected. Gut-derived LPS and proinflammatory mediators can intensify systemic inflammation and impair the BBB; circulating metabolic and inflammatory signals can influence the hypothalamus through the ARC-ME interface; and persistent inflammation combined with barrier dysfunction can create conditions that favor local hypothalamic neuroimmune responses. Ultimately, these pathways allow adipose tissue-derived, lipotoxic, and gut-derived signals to converge on POMC, AgRP/NPY, kisspeptin, and GnRH-related networks, forming an intermediate link through which obesity-associated metaflammation may influence pubertal onset.

## 6 Candidate mechanisms linking hypothalamic neuroimmune remodeling to premature HPG axis activation

**Figure 5:**
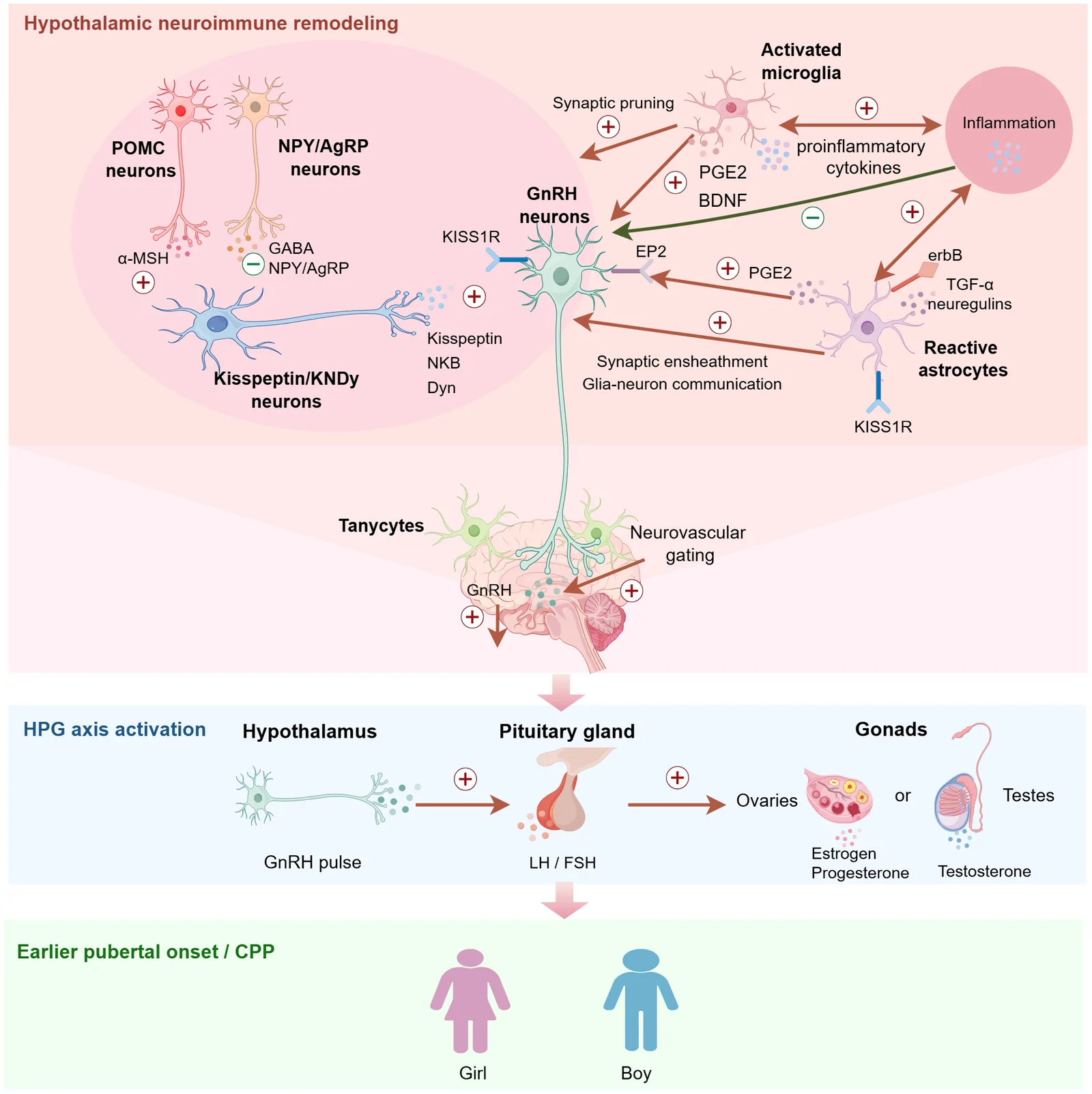
Candidate mechanisms by which hypothalamic neuroimmune remodeling contributes to premature HPG axis activation. After peripheral immunometabolic signals are sensed by the hypothalamus, the neuro-glial-vascular microenvironment in the ARC-ME region may undergo remodeling and influence the timing of kisspeptin–GnRH network activity and HPG axis activation through three mechanisms: microglial activation may release mediators such as proinflammatory cytokines, PGE2, and BDNF; reactive astrocytes may regulate neuronal excitability through gliotransmitter release and altered synaptic ensheathment; and tanycytes may regulate the accessibility of GnRH terminals to hypophyseal portal capillaries through the ME neurovascular interface. Together, these processes may alter functional coupling among POMC, AgRP/NPY, kisspeptin, and GnRH neurons, lower the activation threshold of the HPG axis, and thereby promote earlier pubertal onset or CPP. AgRP, agouti-related peptide; ARC, arcuate nucleus; BDNF, brain-derived neurotrophic factor; CPP, central precocious puberty; FSH, follicle-stimulating hormone; GnRH, gonadotropin-releasing hormone; HPG, hypothalamic–pituitary–gonadal axis; LH, luteinizing hormone; ME, median eminence; NPY, neuropeptide Y; PGE2, prostaglandin E2; POMC, pro-opiomelanocortin. Image created by Figdraw.

Once peripheral immunometabolic signals reach the hypothalamus through the pathways described above, a central question is how they reshape the local neuroimmune microenvironment and alter the timing of kisspeptin-GnRH network activity and HPG axis activation. The hypothalamic microenvironment comprises neurons, glial cells, vascular endothelial cells, pericytes, and ependymal-like cells. Among these populations, microglia, astrocytes, and tanycytes are particularly relevant to immune surveillance, glia-neuron communication, regulation of the neurovascular interface, and GnRH neurosecretion (38). Under conditions of obesity or exposure to diets high in fat and sugar, these glial populations may respond to metabolic, inflammatory, and hormonal signals and modulate GnRH-related networks through cytokines, prostaglandins, neurotrophic factors, gliotransmitters, synaptic remodeling, and neurovascular plasticity (174, 201–203). These processes should be interpreted as candidate regulatory mechanisms that may interact with energy sufficiency, leptin and insulin signaling, adipokines, gut-derived metabolites, sex steroid feedback, and genetic susceptibility, rather than as established independent causal pathways (11, 38). The following sections examine this hypothesis in the context of microglial activation, astrocyte-mediated glia-neuron communication, and tanycyte-regulated neuroendocrine gating (**Figure 5**).

### 6.1 Microglial activation and local inflammatory amplification

Microglia are the resident immune cells of the central nervous system and play key roles in brain development, synaptic remodeling, immune surveillance, and tissue repair (204–206). Recent studies have moved beyond simplistic binary classifications such as “resting/activated” and “M1/M2,” instead emphasizing a continuum of microglial states, regional heterogeneity, and microenvironment-dependent plasticity (207–209). Under physiological conditions, microglia continuously survey the local microenvironment through their dynamic processes and contribute to the sculpting of neural networks through cell-cell contact, soluble factor release, synaptic engulfment, and neuron–microglia signaling (210–214). These properties suggest that microglia are not merely effectors of inflammatory responses but may also participate in the developmental remodeling of hypothalamic neuroendocrine networks around puberty.

Relatively direct experimental evidence links microglia to reproductive axis regulation. Recent findings indicate that hypothalamic microglia may contribute to GnRH neuronal function and HPG axis regulation through RANK signaling. Microglia-specific Rank deficiency can impair microglial activation and alter ME morphology, thereby reducing microglial contact with and engulfment of GnRH nerve terminals. These changes impair GnRH neuronal responsiveness to kisspeptin and ultimately cause GnRH neuronal dysfunction and hypogonadotropic hypogonadism, with consequent disruption of pubertal development and sexual maturation (20). This evidence suggests that microglia are not merely passive contributors to reproductive dysfunction under pathological conditions but may serve as non-neuronal regulatory nodes that maintain GnRH neuronal function and support pubertal development.

In the context of obesity or exposure to diets high in fat and sugar, microglia can sense metabolic and inflammatory signals from peripheral and local sources (174, 203, 215). Circulating saturated fatty acids, LPS, proinflammatory cytokines, and adipokines may influence the hypothalamic microenvironment through the ARC-ME interface or BBB dysfunction. Microglia express receptors involved in lipid and inflammatory sensing, including TLR4, CD36, and triggering receptor expressed on myeloid cells 2 (TREM2), which may translate lipotoxic and inflammatory inputs into local immune responses (206, 216–219).

Activated microglia can release mediators such as IL-1β, IL-6, TNF-α, and prostaglandins, amplify local hypothalamic inflammation, and influence GnRH neuronal function through direct and indirect pathways (24, 220, 221). Regarding direct effects, some GnRH neurons may express receptors or signaling components responsive to inflammatory and stress signals, including IL-1 receptor-related molecules, TNF receptors, IL-6-related signaling components, and the PGE2 receptor EP2. These findings suggest that inflammatory and lipid mediators may directly alter GnRH neuronal excitability, gene expression, and neurosecretory responses (220, 222). Among these mediators, PGE2 is a relatively well-characterized glia-derived signal that promotes GnRH activity and can facilitate GnRH neuronal depolarization and firing through EP2 (19). By contrast, TNF-α can act directly on GnRH neurons through pathways such as TNFR1 and may downregulate KISS1R expression, thereby attenuating kisspeptin-induced activation of GnRH neurons (223).

Regarding indirect effects, cytokines such as IL-1β, IL-6, and TNF-α may act on astrocytes, microglia, kisspeptin/KNDy neurons, and metabolic-sensing neurons such as POMC and AgRP/NPY neurons. These actions may alter kisspeptin inputs, the excitatory-inhibitory balance of the GnRH pulse-generating network, and the local neurosecretory microenvironment (224, 225). For example, IL-1β, IL-1α, TNF-α, and related cytokines can induce astrocytic COX-2/PGE2 production and thereby influence GnRH neuronal activity indirectly through glia-neuron communication (226, 227). In addition, microglia-derived BDNF may regulate neuronal excitability, synaptic plasticity, and GnRH expression and secretion through TrkB and downstream signaling pathways, including PI3K/Akt and MAPK/ERK. This mechanism may provide an additional pathway through which the activation threshold of the HPG axis changes under obesity-associated inflammatory conditions (14, 228). Inflammatory and glia-derived mediators should therefore not be interpreted as uniformly stimulatory signals for GnRH neurons. Their net effects on GnRH function and HPG axis activity are likely to be context dependent and bidirectional, varying with mediator type, inflammatory intensity and duration, developmental stage, sex steroid milieu, hypothalamic region, and the balance between direct effects on GnRH neurons and indirect regulation through kisspeptin/KNDy neurons, metabolic-sensing neurons, and glial cells.

Taken together, microglial activation may influence the HPG axis by sensing immunometabolic signals, amplifying local inflammation, releasing mediators such as PGE2 and BDNF, and remodeling synaptic or functional coupling among POMC/AgRP, kisspeptin, and GnRH-related neurons (20, 229). However, most available evidence is derived from animal models and mechanistic studies. Microglial activation should therefore be regarded as a candidate mechanism linking peripheral metaflammation to hypothalamic inflammatory amplification and an altered threshold for HPG axis activation, rather than as an established causal driver of childhood obesity-associated CPP.

### 6.2 Astrocytes and glia-neuron communication

Astrocytes are an important component of the hypothalamic neuroimmune microenvironment and a key glial population linking metabolic signals, inflammatory responses, and neuroendocrine output (230, 231). Their functions include neurotransmitter clearance, metabolic substrate supply, maintenance of ionic homeostasis, synaptic ensheathment, BBB regulation, and gliotransmitter release (232, 233). Within the hypothalamus, astrocytes are spatially and functionally coupled to GnRH neurons, kisspeptin neurons, and metabolic-sensing neurons such as POMC/AgRP neurons, enabling them to relay local metabolic and inflammatory signals to the regulatory circuits of the reproductive axis (40, 234, 235).

During pubertal onset, astrocytes may promote the functional maturation of GnRH neurons through several glia-neuron communication mechanisms. Astrocytes can release epidermal growth factor family ligands, including transforming growth factor-α (TGF-α) and neuregulins, which activate astrocytic erbB receptors and induce PGE2 synthesis and release. PGE2 subsequently acts on EP2 receptors expressed by GnRH neurons, enhancing their electrical activity and promoting GnRH release (19, 236). Synaptic cell adhesion molecule 1 (SynCAM1), contactin–receptor-type protein tyrosine phosphatase β (RPTPβ), glutamate, and the PGE2–EP2 pathway collectively form a multichannel communication network among astrocytes, GnRH neurons, and their upstream regulatory neurons (237–239). These findings suggest that astrocytic involvement in pubertal onset is not limited to inflammatory responses but encompasses multiple mechanisms, including developmental glial support, synaptic modulation, and facilitation of neurosecretion.

The structural relationship between astrocytes and GnRH neurons is also developmentally regulated. Studies have shown that, during infancy, GnRH neurons can recruit astrocyte progenitors through prostaglandin D2 (PGD2)/DP1 signaling and promote their differentiation into mature astrocytes, thereby establishing the glial microenvironment required for sexual maturation. Disruption of DP1 signaling can impair the GnRH neuronal network and delay pubertal onset (240). These findings support bidirectional communication between astrocytes and GnRH neurons and suggest that this interaction helps prepare the reproductive axis during the prepubertal period.

In obesity or overnutrition, astrocytes may transition from a homeostatic, supportive phenotype to a reactive state. Exposure to a high-fat diet can induce reactive astrogliosis in the hypothalamus, activate inflammatory pathways such as IκB kinase β (IKKβ)/NF-κB, and contribute to hypothalamic inflammation, impaired leptin and insulin signaling, and disrupted energy homeostasis (241). Animal studies further suggest that high-fat diet exposure may be accompanied by increased contact between astrocytic processes and GnRH neurons and enhanced glial fibrillary acidic protein (GFAP) immunoreactivity in the ARC-ME region. These changes may alter the local excitatory milieu surrounding GnRH neurons (19). Astrocytes can also respond to reproductive neuropeptide signals such as kisspeptin, suggesting that they may participate in feedback regulatory loops within the kisspeptin-GnRH network (40). Current evidence indicates that astrocytic responses induced by obesity or high-fat diets may regulate GnRH neuronal excitability and the threshold for HPG axis activation through PGE2, erbB-related signaling, altered synaptic ensheathment, and local inflammatory amplification. However, their causal role in childhood obesity-associated earlier pubertal onset remains to be established through cell-specific interventions and longitudinal population-based studies.

### 6.3 Tanycytes and the neuroendocrine interface

Tanycytes are specialized ependymoglial cells located at the floor of the third ventricle and within the ME. Their cell bodies contact the cerebrospinal fluid, whereas their long processes extend through the ME parenchyma to the perivascular space. This distinctive anatomical organization enables tanycytes to interact simultaneously with the cerebrospinal fluid, hypothalamic neuronal terminals, vascular endothelium, and circulating signals. They are therefore regarded as a key interface linking peripheral metabolic status, hypothalamic neuroendocrine networks, and hypophyseal portal output (158, 242).

The ME is particularly important in regulating GnRH neurosecretion. GnRH neuronal cell bodies are located primarily in the hypothalamic-preoptic regions, whereas their axons project to the ME, where GnRH is released into the hypophyseal portal system. Tanycytic end-feet can dynamically ensheath GnRH nerve terminals, forming a plastic physical barrier. Retraction of these end-feet provides GnRH nerve terminals with greater access to the perivascular space and portal capillaries, thereby facilitating the entry of GnRH into the hypophyseal portal circulation (35). Coordinated signaling between glial and endothelial cells can stimulate tanycytic PGE2 release, which promotes end-foot retraction and facilitates GnRH secretion into the hypophyseal portal circulation (36, 158). Tanycytes are therefore not passive structural scaffolds but active regulators of plasticity at the GnRH neurovascular interface.

Tanycytes may also function as key transduction nodes between metabolic signals and the reproductive axis. Studies have shown that tanycytes in rodents and humans express estrogen receptor α (ERα) and that its expression and function are associated with reproductive status. Tanycytic ERα signaling may contribute to the estrogen-mediated regulation of pulsatile GnRH/LH release, fasting-induced NPY neuronal activity, and feeding responses (243). These findings suggest that tanycytes can integrate sex steroid feedback, nutritional status, and neuroendocrine output. In obesity, circulating adipokines, insulin, free fatty acids, inflammatory mediators, and gut-derived signals may influence tanycytic barrier function, transport capacity, and end-foot plasticity through the ARC-ME interface, thereby modulating hypothalamic sensitivity to peripheral energy status and inflammatory burden (244).

Compared with the evidence available for microglia and astrocytes, direct evidence implicating tanycytes in childhood obesity or CPP remains limited. Current studies more strongly support their physiological role as gatekeeping structures at the neuroendocrine interface than demonstrate specific pathological alterations in obesity-associated earlier pubertal onset. In this review, tanycytes are therefore considered a candidate structural mechanism through which peripheral metaflammation may influence hypothalamic access to circulating signals, the proximity of GnRH terminals to portal vessels, and the efficiency of GnRH release.

### 6.4 An integrated hypothesis of hypothalamic neuroimmune remodeling in HPG axis regulation

Collectively, obesity-associated hypothalamic neuroimmune remodeling may influence HPG axis activation at three interconnected levels. First, inflammatory and lipid mediators released by microglia and astrocytes, including IL-1β, IL-6, TNF-α, PGE2, and BDNF, may alter GnRH neuronal excitability and the neurosecretory microenvironment surrounding GnRH neurons and their upstream regulatory networks. Second, microglial synaptic remodeling and astrocytic process ensheathment may modify functional coupling among POMC/AgRP, kisspeptin, and GnRH-related neurons, thereby affecting the excitatory-inhibitory balance of the GnRH pulse-generating network. Third, tanycytic and ME neurovascular plasticity may regulate the access of circulating signals to the hypothalamus and the efficiency of GnRH release into the hypophyseal portal system (**Table 2**). Inflammatory inputs should therefore be interpreted as context-dependent modulators rather than uniformly stimulatory signals. Their net effects on HPG axis activation are likely determined by inflammatory intensity and duration, developmental stage, and the cellular targets involved.

**Table 2:** Candidate glial mechanisms in hypothalamic neuroimmune remodeling and HPG-axis regulation.

Glial cells	Signals, receptors, or pathways	Candidate neuroimmune remodeling mechanism	Potential impact on HPG axis	Evidence source	References
Microglia	RANK signaling	RANK deficiency reduces microglial interactions with GnRH terminals and impairs GnRH neuronal responsiveness to kisspeptin.	Supports GnRH neuronal function and normal pubertal development.	Human and animal studies	(20)
	TLR4, CD36, TREM2	Microglia sense lipotoxic and inflammatory signals and convert them into local immune responses.	May alter the inflammatory and neurosecretory milieu of GnRH-related networks.	Animal and cell-based studies	(215–219)
	IL-1β, IL-6, TNF-α; PGE2–EP2; BDNF–TrkB	Cytokines, prostaglandins, and neurotrophic factors directly or indirectly regulate GnRH neurons and upstream networks.	Context-dependent stimulation or inhibition of GnRH/HPG-axis activity.	Human, animal, and cell-based studies	(220, 222–228)
	Synaptic pruning and network remodeling	Microglial remodeling may alter coupling among POMC/AgRP, kisspeptin, and GnRH-related neurons.	May shift the excitation/inhibition balance of the GnRH pulse-generating network.	Animal studies	(20, 211–214, 229)
Astrocytes	TGF-α, neuregulins; erbB; PGE2–EP2	Astrocytic erbB activation induces PGE2 release, which stimulates GnRH neurons through EP2 receptors.	Enhances GnRH neuronal firing and release.	Animal and cell-based studies	(19, 236)
	SynCAM1; contactin–RPTPβ; glutamate; PGE2	Adhesion molecules and gliotransmitters mediate astrocyte–GnRH communication.	Supports GnRH network integration and neuronal excitability.	Animal and cell-based studies	(19, 239)
	PGD2–DP1 signaling	GnRH-derived PGD2 recruits astrocyte progenitors through DP1 signaling during infancy.	Supports GnRH network maturation and timely pubertal development.	Animal and cell-based studies	(240)
	HFD; IKKβ/NF-κB; GFAP; altered astrocytic apposition	HFD induces reactive astrogliosis and alters astrocytic contacts with GnRH neurons.	May modify GnRH excitability and the HPG-axis activation threshold.	Animal studies	(19, 241)
Tanycytes	Neuroendocrine interface gating; PGE2	Tanycytic end-foot remodeling regulates GnRH terminal access to portal capillaries.	Facilitates GnRH release into the hypophyseal portal circulation.	Animal and cell-based studies	(35, 36, 158)
	ERα signaling	Tanycytic ERα integrates estrogen feedback with hypothalamic metabolic circuits.	Modulates GnRH/LH pulsatility and reproductive–metabolic coupling.	Animal studies	(243)

AgRP, agouti-related peptide; BDNF, brain-derived neurotrophic factor; CD36, cluster of differentiation 36; DP1, prostaglandin D2 receptor 1; EP2, prostaglandin E2 receptor 2; ERα, estrogen receptor alpha; ErbB, Erb-B receptor tyrosine kinase; GFAP, glial fibrillary acidic protein; GnRH, gonadotropin-releasing hormone; HFD, high-fat diet; HPG, hypothalamic–pituitary–gonadal; IKKβ, inhibitor of nuclear factor-κB kinase subunit beta; IL, interleukin; LH, luteinizing hormone; NF-κB, nuclear factor kappa B; PGD2, prostaglandin D2; PGE2, prostaglandin E2; POMC, proopiomelanocortin; RANK, receptor activator of nuclear factor-κB; RPTPβ, receptor-type protein tyrosine phosphatase beta; SynCAM1, synaptic cell adhesion molecule 1; TGF-α, transforming growth factor alpha; TLR4, Toll-like receptor 4; TNF-α, tumor necrosis factor alpha; TREM2, triggering receptor expressed on myeloid cells 2; TrkB, tropomyosin receptor kinase B.

This integrated model positions microglia, astrocytes, and tanycytes as interconnected components of a glia-neuron-vascular communication network rather than independent drivers of pubertal advancement. In obesity, this network may become more responsive to metabolic and inflammatory inputs, potentially lowering the activation threshold of the kisspeptin-GnRH network and HPG axis. However, clinical evidence remains largely observational, and findings from animal models cannot be directly extrapolated to human CPP. Longitudinal pediatric cohorts, single-cell and spatial omics analyses, and cell type-specific experimental approaches are needed to determine whether hypothalamic neuroimmune remodeling represents a causal bridge between childhood obesity and premature HPG axis activation.

## 7 Diagnostic and therapeutic implications

### 7.1 Candidate biomarkers

The diagnosis of CPP currently relies primarily on clinical manifestations, bone-age assessment, evidence of gonadal-axis activation, and the GnRH stimulation test. Although the GnRH stimulation test is used to distinguish among different types of precocious puberty, it is time-consuming, requires repeated blood sampling, and may be poorly tolerated by children. The identification of simple, stable, and reproducible adjunctive biomarkers would therefore be clinically valuable (87). Within the framework in which metaflammation and an imbalanced immune microenvironment contribute to earlier pubertal onset, candidate biomarkers have expanded beyond traditional HPG axis hormones to include neuropeptides, epigenetic regulatory proteins, inflammatory mediators, adipokines, angiogenic factors, and gut microbiota-derived metabolites. These markers may not only support the diagnosis of CPP but also help identify high-risk phenotypes of metabolically associated earlier pubertal onset.

Neuroendocrine and epigenetic biomarkers may reflect the upstream regulatory state of the HPG axis. Kisspeptin and makorin ring finger protein 3 (MKRN3) were among the earliest candidate markers investigated. Studies have shown that girls with CPP have elevated serum kisspeptin levels and reduced MKRN3 levels. Combining these two markers provides some diagnostic utility for CPP but has limited ability to distinguish CPP from premature thelarche (88). Methyl-CpG-binding domain protein 3 (MBD3), an epigenetic regulatory protein associated with MKRN3, is elevated in girls with CPP and correlates with LH, FSH, and ovarian size, suggesting its potential value as an adjunctive diagnostic marker (89). However, these biomarkers require further validation across different ages, sexes, and developmental stages.

Immune cell-related indicators may provide additional information for evaluating the immunometabolic phenotype of obesity-associated CPP. A recent cross-sectional study included healthy controls, children with obesity alone, children with CPP alone, and children with obesity-associated CPP. The obesity-alone and obesity-associated CPP groups had higher inflammation-related hematological indices than the CPP-only group, particularly white blood cell count and the neutrophil-to-lymphocyte ratio (NLR). Obesity-associated CPP was also positively associated with LH, FSH, and other indicators, and receiver operating characteristic (ROC) curve analyses suggested that some inflammatory and metabolic indices had predictive value for CPP among children with obesity (92). In addition, an exercise intervention study showed that girls with CPP exhibited decreases in inflammatory indicators, including TNF-α, white blood cell count, and neutrophil count, after aerobic exercise. These changes were accompanied by improvements in puberty-related indicators, including LH, FSH, uterine length, and ovarian volume. Some indicators rebounded after exercise cessation, suggesting that peripheral inflammatory status may be associated with CPP progression and intervention response (91). Immune cell-derived indicators, including neutrophil and lymphocyte counts, total white blood cell count, and NLR, may therefore serve as adjunctive candidate biomarkers of systemic low-grade inflammation and immunometabolic status in obesity-associated CPP. However, because these indices are susceptible to multiple confounding factors and lack longitudinal validation, they are not currently suitable as independent diagnostic criteria.

Inflammatory mediators and adipokines may reflect the immunometabolic status of children with CPP. Classical inflammatory markers such as IL-6 and CRP are associated with pubertal timing. However, their associations with Tanner breast stage may be attenuated after adjustment for body fat percentage, indicating that inflammatory indices should not be interpreted independently of BMI, adiposity, and metabolic status (52). Clinical studies have shown that girls with CPP have reduced total adiponectin levels, which are negatively correlated with Tanner stage and BMI, whereas high-molecular-weight adiponectin levels are elevated and positively correlated with Tanner stage and LH (79). A meta-analysis further demonstrated that girls with CPP have higher serum leptin levels and lower adiponectin levels than healthy controls (80). Decreased omentin-1 and increased chemerin levels in children with CPP who do not have obesity further suggest that adipokine abnormalities are not simply proxies for obesity but may reflect broader immunoendocrine dysregulation (90). Leptin, adiponectin, and other adipokines may therefore be more appropriately used as adjunctive indicators for the risk stratification of metabolically associated CPP than as standalone diagnostic criteria.

Vascular endothelial growth factor (VEGF), the gut microbiota, and microbial metabolites offer emerging directions for research on nontraditional CPP biomarkers. VEGF-A is elevated in girls with CPP and is associated with advanced bone age, Tanner stage, and estradiol levels, suggesting that it may link inflammation, angiogenesis, and skeletal maturation (97, 245). Current studies also indicate that children with CPP or obesity-associated precocious puberty exhibit alterations in gut microbiota composition and metabolic function. Clinical microbiome studies of girls with obesity-associated precocious puberty suggest that genera such as Dialister, Bacteroides, Bifidobacterium, Collinsella, and Romboutsia may be associated with HPG axis activation (83). Another 16S rRNA sequencing study identified Sellimonas, Intestinibacter, Anaerostipes, the Ruminococcus gnavus group, and Oscillibacter as potential microbial biomarkers of obesity with precocious puberty (86). In addition, a comparative study of children with CPP and overweight suggested that microbiota-induced nitric oxide (NO) synthesis and neurotransmitter-related metabolism may link CPP to obesity (84). Integrated microbiome-metabolomics analyses further identified multiple abnormalities in the gut microbiota and fecal and blood metabolites of children with CPP, suggesting that Streptococcus, the NO synthesis pathway, and steroid-related metabolites may have value as candidate biomarkers (96). A systematic review and meta-analysis identified reproducible alterations in the gut microbiota of individuals with CPP, accompanied by reduced levels of SCFAs such as butyrate and propionate (85). A two-sample Mendelian randomization study further suggested potentially causal associations between microbial taxa such as Bacteroides and Alistipes and the risk of CPP (95). Gut microbiota composition, SCFAs, NO synthesis-related functions, and steroid or neuroactive metabolites may therefore serve as adjunctive candidate biomarkers for CPP and obesity-associated CPP. However, microbiome findings are strongly influenced by diet, age, BMI, geographic region, analytical platform, and sample size. These markers are therefore more appropriately regarded as mechanistic clues and potential sources of noninvasive biomarkers than as direct diagnostic tools.

Given the heterogeneity of CPP, multimarker models may have greater translational potential than any single biomarker. Previous studies have shown that basal LH, insulin-like growth factor 1 (IGF-1), insulin-like growth factor-binding protein 3 (IGFBP-3), and dehydroepiandrosterone sulfate (DHEAs) may support the early identification of CPP, with combined LH and DHEAs testing showing relatively high diagnostic performance (93). A recent serum-based model combining LH, kisspeptin, vitamin D, and estradiol showed favorable performance in identifying CPP, suggesting that integrated strategies incorporating endocrine, nutritional–metabolic, and neuropeptide signals may outperform single-marker approaches (87). Predictive models based on IGF-1 and IGFBP-3 standard deviation scores also highlight the importance of age, sex, and assay standardization (94). Future clinically meaningful efforts should focus not on identifying a single “replacement” marker but on developing integrated predictive models that incorporate age, sex, BMI or body fat percentage, bone age, basal LH, and immunometabolic biomarkers.

### 7.2 Therapeutic prospects

Current evidence suggests that metaflammation and disruption of the hypothalamic immune microenvironment may contribute to the association between childhood metabolic disorders and earlier pubertal onset. Interventions for metabolically associated earlier pubertal onset should therefore not be limited to downstream suppression of the HPG axis but should also address modifiable upstream factors, including obesity, insulin resistance, adipokine dysregulation, low-grade inflammation, and gut dysbiosis (**Table 3**).

**Table 3:** Current and emerging interventions for obesity-associated earlier pubertal onset and CPP.

Interventions	Clinical positioning	Mechanism	Evidence	Limitations	References
GnRHa	Standard therapy for CPP	Suppress HPG-axis activation	Established efficacy in controlling pubertal progression and bone maturation	Does not address upstream metabolic drivers	(246, 247)
Lifestyle and weight management	Foundational upstream management	Reduce adiposity, insulin resistance, and inflammation	Metabolic benefits established; puberty-specific evidence remains limited	Variable adherence and response	(91, 181, 248)
Metformin	Potential adjunct in selected patients	Improve insulin sensitivity and modulate AMPK, mTOR, and inflammatory signaling	Limited pediatric and high-risk population studies	Off-label use and limited generalizability	(249–251)
Anti-inflammatory nutritional strategies	Exploratory metabolic adjunct	Attenuate oxidative and inflammatory signaling	Indirect clinical and mechanistic support	Heterogeneous formulations and limited puberty-specific trials	(252–254)
Microbiota-directed strategies	Emerging adjunct	Modulate dysbiosis, barrier integrity, and microbial metabolites	Mainly preclinical and preliminary clinical evidence	Poor standardization and variable responses	(95, 181)
Central neuroimmune targeting	Preclinical therapeutic concept	Modulate hypothalamic inflammatory and metabolic pathways	Supported by mechanistic and animal studies	No validated clinical targets or safety data	(181, 241, 255, 256)
Traditional Chinese medicine and natural products	Exploratory preclinical adjunct	Multi-target modulation of inflammatory, metabolic, and endocrine pathways	Luteolin improved pubertal and hormonal outcomes in a danazol-induced rat model, possibly via TLR-related signaling	Predominantly preclinical evidence; active constituents, dosing, standardization, safety, and clinical efficacy remain uncertain	(257)

AMPK, AMP-activated protein kinase; CPP, central precocious puberty; GnRH, gonadotropin-releasing hormone; GnRHa, gonadotropin-releasing hormone analogs; HFD, high-fat diet; HPG, hypothalamic–pituitary–gonadal; LPS, lipopolysaccharide; SCFAs, short-chain fatty acids; TLR, Toll-like receptor.

Gonadotropin-releasing hormone analogs (GnRHa) remain the standard treatment for CPP. They primarily suppress premature HPG axis activation, thereby delaying the progression of secondary sexual characteristics, slowing bone maturation, and improving predicted adult height (1, 246, 247). However, GnRHa act mainly on pituitary GnRH receptors and represent a downstream HPG axis-suppression strategy. They do not fully address upstream pathological processes associated with obesity, high-fat diet exposure, and chronic low-grade inflammation (246). In children with CPP who also have overweight, obesity, or overt metabolic abnormalities, GnRHa therapy should therefore be accompanied by weight management, metabolic risk assessment, and lifestyle interventions.

The treatment paradigm for metabolically associated earlier pubertal onset is gradually shifting from symptomatic treatment after HPG axis activation toward intervention during a metabolic window before or early in axis activation. Studies of metformin provide a rationale for drug repurposing within this framework. Mechanistic studies indicate that metformin can modulate glucose metabolism, insulin sensitivity, and inflammatory responses through pathways involving AMPK, mTOR, and NF-κB/IL-6 signaling (249). In children with obesity but without diabetes, metformin has shown beneficial effects on BMI z-score, insulin sensitivity, the adiponectin-to-leptin ratio, and selected inflammatory markers, suggesting that it may be more applicable to children at high risk because of insulin resistance or other metabolic abnormalities (250). A meta-analysis of girls with precocious puberty and low birth weight showed that metformin improved BMI z-score, fat mass, fasting insulin levels, lipid profiles, and androgen-related indices and may reduce the risk of progression toward obesity- or polycystic ovary syndrome-related phenotypes (251). However, the available evidence is largely limited to specific metabolic phenotypes and is insufficient to support metformin as a routine treatment for CPP. Metformin should therefore be regarded as a potential adjunctive strategy for children at high metabolic risk.

Lifestyle modification is the most fundamental and scalable strategy for metabolically associated earlier pubertal onset. Physical activity not only supports weight management but may also complement GnRHa therapy by improving adipokine profiles, insulin sensitivity, and inflammatory status. Studies have shown that, among girls with CPP receiving standard GnRHa therapy, the addition of exercise training increases adiponectin levels and reduces TNF-α, resistin, neutrophil counts, and selected indicators of pubertal progression, suggesting that exercise may improve inflammatory and metabolic status in children with CPP (91, 248). Nutritional strategies, including reducing the consumption of diets high in fat and sugar, increasing dietary fiber intake, optimizing fatty acid composition, and improving overall diet quality, may help reduce the burden of metaflammation (7, 181). Polyphenols, ω-3 fatty acids, and their specialized pro-resolving mediators also have potential to ameliorate chronic low-grade inflammation (252–254). At present, however, these anti-inflammatory nutritional strategies are supported primarily by evidence from metabolic risk management and mechanistic studies, and high-quality intervention trials evaluating CPP-related outcomes remain lacking.

The hypothalamic immune microenvironment is a candidate therapeutic target linking metabolic disorders to HPG axis activation. However, current interventional evidence is derived mainly from animal and cellular studies and remains far from clinical translation. Astrocytic IκB kinase β (IKKβ)/NF-κB signaling contributes to high-fat diet-induced hypothalamic inflammation and impaired energy metabolism. Inhibition of this pathway reduces glial fibrillary acidic protein (GFAP) reactivity and IL-1β and IL-6 expression while improving food intake, energy expenditure, and glucose metabolism (241). In addition, cellular and animal studies have shown that molecular pathways such as the liver kinase B1 (LKB1)-AMPK/forkhead box O1 (FOXO1) axis and Rap1B regulate inflammatory mediators, GnRH/Kiss1 expression, and indices related to gonadal-axis activation (255, 256). These findings suggest that the central immune microenvironment may be modifiable. However, the safety of these approaches, targeted delivery strategies, effects of developmental timing, and long-term neuroendocrine consequences require systematic evaluation.

The gut microbiota represents another potentially modifiable target for metabolically associated earlier pubertal onset. Existing reviews suggest that the gut microbiota may influence HPG axis function through mechanisms involving β-glucuronidase-mediated enterohepatic circulation of estrogens, SCFA metabolism, LPS-mediated inflammatory pathways, leptin and insulin balance, and epigenetic modifications (51). Probiotics, diets lower in sugar and higher in fiber, and microbiota-targeted natural products may therefore represent future adjunctive strategies. However, randomized controlled trials are required to determine their effects on pubertal timing, HPG axis indices, and long-term metabolic outcomes. Traditional Chinese medicine and natural products may modulate inflammatory, metabolic, and endocrine networks through multitarget mechanisms and therefore have exploratory potential as adjunctive therapies for CPP. Nevertheless, current evidence is derived primarily from animal experiments and network pharmacology studies. Active components, dosage, safety, and clinical efficacy must be clarified before standardized clinical application can be considered (257).

Future interventions should adopt a stratified management approach. For children with confirmed CPP, GnRHa remain the foundational therapy. For those with overweight or obesity, insulin resistance, or chronic low-grade inflammation, weight management, exercise interventions, dietary optimization, and metabolic risk monitoring should be implemented concurrently. Metformin, microbiota modulation, anti-inflammatory nutritional strategies, and interventions targeting the central immune microenvironment remain promising but should be considered exploratory until supported by stronger evidence from pediatric intervention studies.

## 8 Conclusion

The associations of childhood obesity with earlier pubertal onset and CPP are supported by clinical and epidemiological evidence. Obesity should not be viewed merely as excess energy storage but as an immunometabolic state shaped by adipose tissue dysfunction, insulin resistance, adipokine dysregulation, gut dysbiosis, and chronic low-grade inflammation. Peripheral metaflammation may reach or influence the hypothalamus through the ARC-ME complex, BBB dysfunction, and the gut-brain axis, thereby affecting the hypothalamic metabolic-reproductive network through glia-neuron-vascular communication.

Within this framework, microglia, astrocytes, and tanycytes may alter kisspeptin-GnRH network activity and the threshold for HPG axis activation through inflammatory amplification, gliotransmitter-mediated modulation, synaptic remodeling, and gating at the neuroendocrine interface. However, these remain candidate pathways rather than established causal mechanisms in human CPP. Future studies should integrate longitudinal pediatric cohorts, immunometabolic profiling, single-cell and spatial omics, and cell type-specific mechanistic validation to clarify the developmental timing, sex specificity, and causal relevance of hypothalamic neuroimmune remodeling.

Clinically, this framework supports the development of multidimensional risk-stratification models integrating endocrine, anthropometric, metabolic, inflammatory, adipokine, and gut microbiota-related indicators. GnRHa remain the standard treatment for CPP, but the management of children with obesity-related metabolic risk should also address modifiable upstream factors, including weight status, insulin resistance, chronic inflammation, diet quality, and physical activity. Such an approach may broaden CPP management beyond downstream suppression alone to include earlier risk identification and integrated metabolic-endocrine prevention.
